# NO‐Driven Janus Nanomotor Enhances T‐Cell Infiltration by Reconstructing Tumor‐Associated Blood and Lymphatic Vessels

**DOI:** 10.1002/advs.202512090

**Published:** 2025-09-08

**Authors:** Qingping Ma, Shunli Fu, Yiming Xia, Shuying Gao, Zhenxing Xia, Panpan Gu, Shijun Yuan, Jinhu Liu, Shuang Liang, Nan Li, Qinglin Yang, Weiwei Mu, Jie Liu, Xinrui Liu, Yongjun Liu, Na Zhang

**Affiliations:** ^1^ Department of Pharmaceutics Shandong Key Laboratory of Targeted Drug Delivery and Advanced Pharmaceutics NMPA Key Laboratory for Technology Research and Evaluation of Drug Products Key Laboratory of Chemical Biology (Ministry of Education) State Key Laboratory of Discovery and Utilization of Functional Components in Traditional Chinese School of Pharmaceutical Sciences Cheeloo College of Medicine Shandong University 44 Wenhuaxi Road Jinan Shandong 250012 China

**Keywords:** in situ cancer vaccine, nanomotor, nitric oxide, T‐cell infiltration, tumor blood vessel normalization

## Abstract

The effectiveness of antitumor immunotherapy is limited to immune cell infiltration into solid tumors, primarily via T‐cell migration through tumor blood vessels. This study introduces a multifunctional nitric oxide (NO)‐driven hollow gold Janus nanomotor (HAM) designed to promote tumor blood vessel normalization and increase T‐cell infiltration, thereby enhancing the immune response against tumors. It is revealed that self‐generated NO facilitates the penetration of HAM into tumors and increases pericyte coverage of blood vessels, thereby enhancing intratumoral T‐cell infiltration. HAMs then induce and capture whole‐tumor antigens to enhance T‐cell activation as an in situ cancer vaccine. Additionally, vascular endothelial growth factor C (VEGFC) is used in combination to induce functional lymphangiogenesis, aiding dendritic cell (DC) migration of tumor‐draining lymph nodes (TDLNs). In B16F10 mice, the proportion of tumor‐infiltrating T cells increased from 0.5% to 27.4% while that of mature DCs in TDLNs increased from 4.3% to 16.6%, markedly improving tumor‐killing effects. Similar outcomes are observed in 4T1 tumor‐bearing mice. Collectively, this study highlights the importance of paving the way for intratumoral infiltration of immune cells via nanomotors, which provides a novel idea for enhancing antitumor immunotherapeutic effects.

## Graphical abstract

NO‐driven hollow gold Janus nanomotors (HAM) are engineered to improve tumor blood vessel normalization throughout the entire tumor region to enhance T‐cell intratumoral infiltration, and generate and capture whole‐tumor antigens as an in situ cancer vaccine to enhance T‐cell activation, therefore enhancing the antitumor immune response.

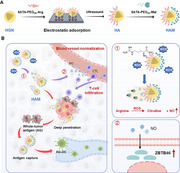

## 1 Introduction

Immune cell infiltration is a key factor limiting antitumor immunotherapy efficacy.^[^ ^1^, ^2^ ^]^ For instance, CAR‐T cells face challenges in solid tumors due to insufficient infiltration.^[^ ^3^ ^]^ Tumor blood vessels are the main pathway for activated T cells to infiltrate into the interior of tumors.^[^ ^4^ ^]^ Unfortunately, tumor‐associated blood and lymphatic vessels collapse due to the compression exerted by tumor cells, resulting in vascular leakage and impaired lymphatic drainage. Imbalanced proangiogenic and antiangiogenic factors at tumor sites lead to rapid yet abnormal blood vessel formation that supports tumor growth and impedes immune effector cell infiltration.^[^ ^5^, ^6^, ^7^, ^8^ ^]^ Hence, normalizing tumor blood vessels—rather than inhibiting their formation—has become an important area of research,^[^ ^9^ ^]^ as it can improve vessel function without increasing the risks of hypertension and arterial thromboembolic events caused by traditional antiangiogenic therapies.^[^ ^10^ ^]^ Nitric oxide (NO) has different blood vessel‐related biological functions.^[^ ^11^, ^12^ ^]^ Low‐dose NO can normalize tumor blood vessels and enhance pericyte coverage, thereby creating a favorable microenvironment for T‐cell infiltration and survival and enhancing immunotherapy outcomes.^[^ ^13^, ^14^ ^]^ Thus, combining NO‐mediated tumor blood vessel normalization with immunotherapy may maximize immune cell infiltration and therapeutic delivery to tumors.

To enable T cells to infiltrate tumors and exert cytotoxic effects, abnormal blood vessel function must be restored. High interstitial and osmotic pressures in tumors hinder the deep penetration of nano‐drug delivery systems, resulting in their accumulation in the superficial regions of tumors and limiting their effectiveness.^[^ ^15^, ^16^, ^17^ ^]^ Micro/nanomotors, with the ability to move autonomously, have great advantages in breaking through biological barriers in tumors.^[^ ^18^, ^19^ ^]^ Among them, gas‐driven nanomotors have the advantage of being specifically driven by the endogenous tumor environment.^[^ ^20^, ^21^, ^22^ ^]^ NO not only acts as a blood vessel reconstructing molecule but also may operate as an endogenous nanomotor driver to promote deep penetration.^[^ ^23^ ^]^ The application of NO‐driven nanomotors in drug delivery has been explored in recent studies.^[^ ^24^, ^25^ ^]^ Hence, we aimed to construct a Janus NO‐driven nanomotor capable of deeply penetrating into tumor sites to promote tumor blood vessel normalization throughout the entire tumor region, while simultaneously generating and capturing whole‐tumor antigens. By achieving these functions, the Janus NO‐driven nanomotor further induces T‐cell activation and infiltration, thereby exerting robust antitumor immune effects as a novel in situ cancer vaccine.

**Figure 1:**
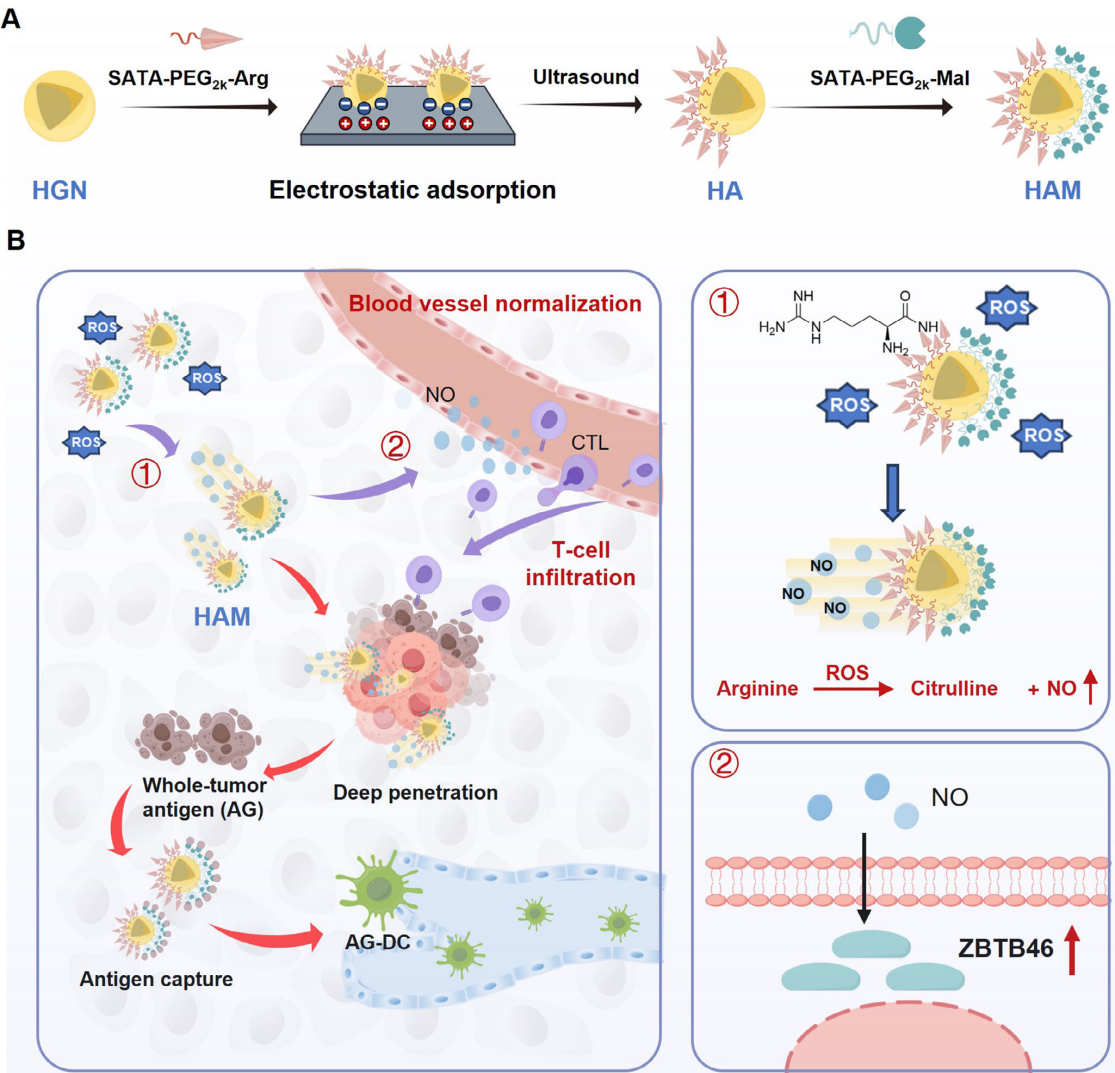
Schematic of NO‐driven hollow gold Janus nanomotor (HAM) construction and capacity to deeply penetrate tumors and reconstruct abnormal tumor blood vessels for enhanced T‐cell infiltration. A), NO‐driven hollow gold Janus nanomotors (HAMs) consist of hollow gold nanospheres (HGNs) as the core, asymmetrically modified with SATA‐PEG_2000_‐Arginine (Arg) and SATA‐PEG_2000_‐Maleimide (Mal) via electrostatic adsorption. B), HAMs penetrate tumors driven by NO, reconstruct abnormal tumor blood vessels, and capture whole‐tumor antigens under laser irradiation to promote T‐cell activation and infiltration, initiating a robust antitumor immune response. Created with Figdraw.com.

In this study, NO‐driven hollow gold Janus nanomotors (HAM) were novelly engineered to reconstruct abnormal blood vessels throughout the whole tumor region for enhanced T‐cell infiltration (**Figure** **1**). HAM was loaded in RGD‐targeted temperature‐sensitive nanoparticles (RN) to construct HAM‐RN, which could target the tumor and release HAM under laser irradiation. HAM consisted of hollow gold nanospheres as the core and asymmetrically modified with L‐arginine (Arg) and maleimide (Mal). The resulting structure could generate reactive oxygen species (ROS) from hollow gold nanospheres under laser irradiation and react with Arg to generate NO, thus overcoming the high intratumoral pressure and promoting deep penetration of HAM as the driving force. The production of ROS and the photothermal therapy (PTT)‐mediated immunogenic cell death (ICD) effect promoted whole‐tumor antigen generation. Mal modified on the surface of HAM would then capture whole‐tumor antigens, enhancing antigen presentation by DCs. The generated NO reconstructed abnormal tumor blood vessels to improve the infiltration of activated T cells into tumors. Furthermore, Vascular endothelial growth factor C (VEGFC), which could activate vascular endothelial growth factor receptor 3 (VEGFR3) to promote lymphangiogenesis, was used in combination with HAM to facilitate the reconstruction of tumor‐associated lymphatic vessels, thus to enhance the migration of DCs to TDLNs for better T‐cell activation. Overall, this study highlighted the importance of paving the way to improve immune cell migration to tumors, providing a novel idea for the enhancement of antitumor immunotherapeutic effects.

## 2 Results and Discussion

### 2.1 HAMs with Hollow Structure Enhance the Photothermal Conversion Capacity and Promote NO‐Driven Movement via the Janus Structure

**Figure 2:**
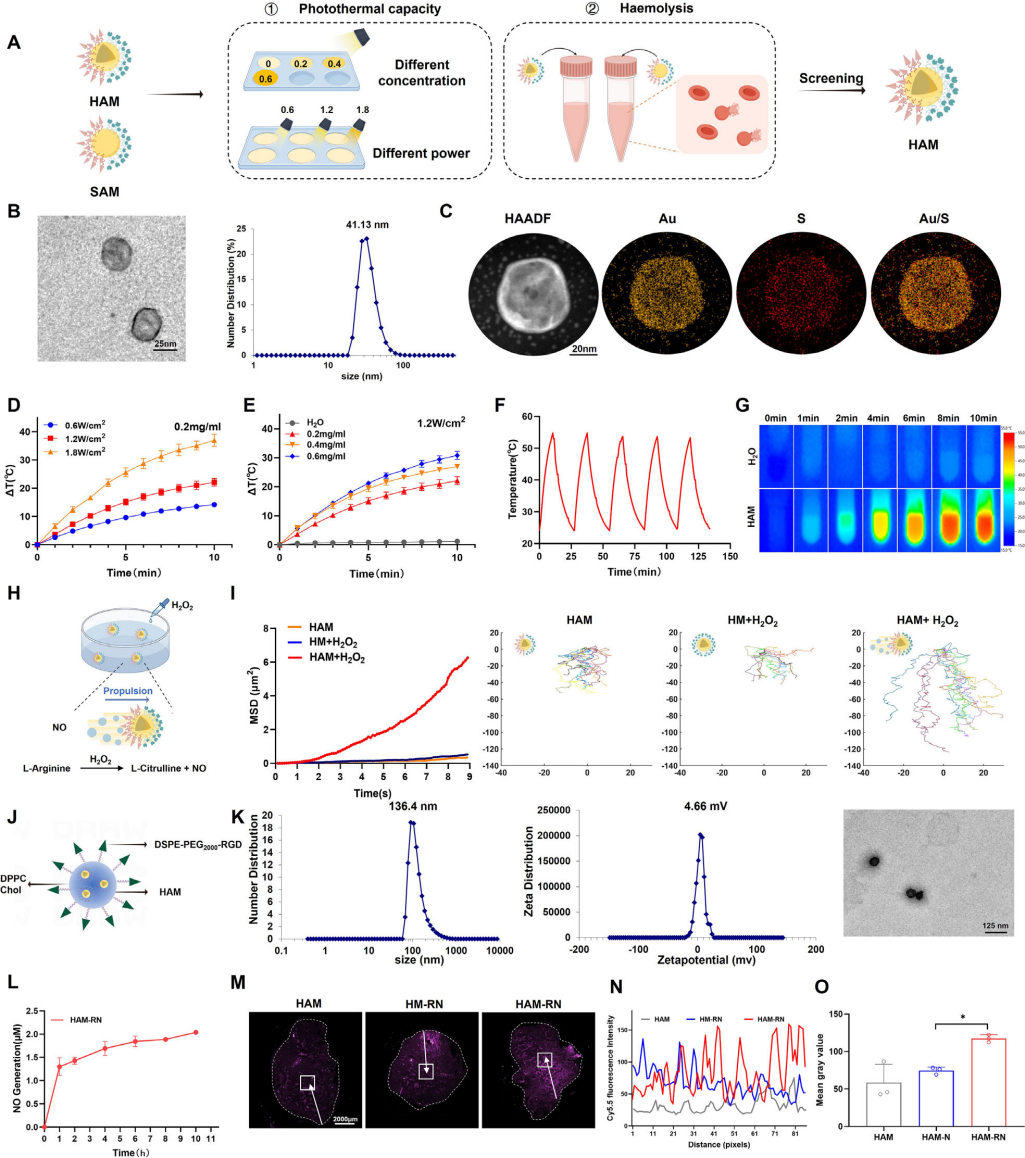
HAMs with hollow structure enhance the photothermal conversion capacity and have the ability of NO‐driven movement via the Janus structure, and HAM‐RNs display great accumulation and deep penetration ability at tumor sites. A), Schematic illustration of the SAM and HAM screening processes. B), Particle size and morphological characterization of HAM. C), STEM‒EDS elemental mapping analysis of HAM. D,E), Temperature profiles of HAM at different powers (D) and different concentrations (E). F), Temperature profile of HAM recorded after five cycles of laser irradiation on/off. G), Infrared thermal imaging of water and HAM (0.2 mg mL^−1^) at different time points under laser irradiation. H), Motion principle of HAM in H_2_O_2_ solution. I), Representative track and MSD fitting lines of HAM, HM+H_2_O_2,_ and HAM+H_2_O_2_ in 90 frames. (n = 10) J), Structural schematic of HAM‐RN. K), Particle size, zeta potential, and morphology of HAM‐RN. L), The generation profile of NO generated by HAM‐RN in H_2_O_2_ solution after laser irradiation. M), Representative confocal images of HAM, HM‐RN, and HAM‐RN accumulated in tumor sections. Scale bar: 2000 µm. N), Cy5.5 fluorescence intensities of HAM, HM‐RN, and HAM‐RN penetrated deeply into tumors. O), Quantification of HAM, HM‐RN, and HAM‐RN accumulation in tumor sections. All the experiments were conducted with three independent replicates. The results are expressed as the mean±SD. One‐way ANOVA analysis of variance with Tukey's post hoc test was used for analysis of statistical significance. ^*^ *p* < 0.05. HM: HAM without Arg modification.

Solid gold nanoparticles (SGNs) modified with Arg and Mal asymmetrically (SAMs) and hollow gold nanospheres (HGNs) modified with Arg and Mal asymmetrically (HAMs) were generated and evaluated for photothermal conversion abilities and hemolytic safety (**Figure** **2**A). SGNs were synthesized by sodium citrate reduction. HGNs were produced through cobalt nanoparticle templating followed by gold shell formation and cobalt core oxidation. Within the UV–Vis absorption spectra, SGNs and HGNs had maximum absorption peaks at 518 and 813 nm, respectively, indicating a superior photothermal conversion capacity of HGN in the near infrared (NIR) region (Figure S1A, Supporting Information). The particle sizes were ≈ 27.9 ± 1.86 nm for SGNs and 31.5 ± 3.67 nm for HGNs. Transmission electron microscopy (TEM) confirmed their solid and hollow spherical morphologies (Figure S1B,C, Supporting Information).

The functionally modified materials N‐Succinimidyl S‐Acetylthioglycolate (SATA)‐PEG_2000_‐Arg and SATA‐PEG_2000_‐Mal were successfully synthesized. Their chemical structures were characterized by ^1^H nuclear magnetic resonance (^1^H NMR; Figure S2A,B, Supporting Information). The functional materials included sulfhydryl groups protected by SATA, which could undergo deprotection through reaction with hydroxylamine hydrochloride, thereby enabling their attachment to SGN and HGN surfaces via gold‒sulfur bonds to produce SAM and HAM. Asymmetric modification of the two types of gold nanoparticles (AuNPs) was achieved by sequentially modifying SATA‐PEG_2000_‐Arg and SATA‐PEG_2000_‐Mal through positively charged silanized glass‐mediated electrostatic adsorption. Post‐modification, the particle sizes of SAM and HAM increased to 39.4 ± 3.23 nm and 41.5 ± 0.48 nm, respectively, with a modification layer observable on both surfaces (Figure 2B; Figure S1D, Supporting Information). Energy dispersive spectroscopy (EDS) analysis identified overlapping regions of sulfur and gold, indicating successful formation of gold‐sulfur bonds on the SGN and HGN surfaces (Figure 2C; Figure S1E, Supporting Information). These findings confirmed that activated SATA‐PEG_2000_‐Arg and SATA‐PEG_2000_‐Mal were successfully modified on the surfaces of both types of gold nanoparticles.

After synthesizing SAM and HAM, the photothermal conversion performance of the two AuNPs was investigated under 808 nm NIR laser irradiation. At 0.4 mg mL^−1^, SAM's relative temperature increased to 7.37±0.61, 12.3±0.49 °C, and 26.0 ± 0.79 °C within 10 min at 0.6, 1.2, and 1.8 W cm^−2^, respectively (Figure S3A, Supporting Information), and by 8.3 ± 0.05, 12.3 ± 0.49, and 17.2 ± 0.66 °C over 10 min at 0.2, 0.4, and 0.6 mg mL^−1^, under 1.2 W cm^−2^, respectively (Figure S3B, Supporting Information). In contrast, the relative temperatures of HAM (0.2 mg mL^−1^) increased by 14.2 ± 0.24, 22.1 ± 1.11, and 37.0 ± 1.74 °C at 0.6, 1.2, and 1.8 W cm^−2^ (10 min), respectively, and by 22.1 ± 1.11, 27.0 ± 0.25, and 30.8 ± 1.13 °C over 10 min at 0.2, 0.4, and 0.6 mg mL^−1^, under 1.2 W cm^−2^, respectively (Figure 2D,E). These results were supported by infrared thermal imaging (Figure 2G; Figure S3C, Supporting Information), demonstrating the superior photothermal conversion efficiency of HAM. Both AuNPs maintained stable photothermal conversion after five laser cycles (Figure 2F; Figure S3D, Supporting Information).

In vitro hemolysis assays revealed no discernible (< 5%) hemolysis in the HAM groups within the experimental concentration range (1.56–100 µg mL^−1^). In contrast, ≥ 50 µg mL^−1^ SAM treatment induced hemolysis rates (HRs) > 5%. Thus, HAM demonstrated reduced interaction with blood components and superior biocompatibility and was suitable for intravenous administration. Accordingly, 0.2 mg mL^−1^ HAM at 1.2 W cm^−2^ was selected for subsequent experiments due to superior efficacy and safety (Figure S4, Supporting Information).

The Arg‐functionalized HAM generated NO responsively in the tumor microenvironment (TME) via reactions with ROS, powering its movement. H_2_O_2_ was used to simulate ROS‐rich TME in vitro (Figure 2H). The Janus properties and motility of HAM were assessed via nanoparticle tracking analysis (NTA). The linear fit of the mean‐square displacement (MSD) curve revealed the oriented movement of HAM in H_2_O_2_ solution (Movie S1, Supporting Information), with HM (HAM without Arg) in H_2_O_2_ and HAM in double‐distilled water exhibiting only Brownian motion (Figure 2I; Movies S2 and S3, Supporting Information). Confocal laser scanning microscopy (CLSM) and flow cytometry further assessed the uptake efficiency of FITC‐labeled HAM by B16F10 cells in the HAM, HM+H_2_O_2_, and HAM+H_2_O_2_ groups. The HAM+H_2_O_2_ group exhibited the highest FITC fluorescence intensity among all groups, indicating a higher rate of uptake by B16F10 cells (Figure S5A,B, Supporting Information). Additionally, HAM generated NO continuously for 10 h in H_2_O_2_ solution (Figure S5C, Supporting Information), confirming that its autonomous motion was powered by the NO produced by Arg in ROS‐rich environments_._ Finally, we incorporated Matrigel into the upper chamber of the transwell system to simulate the ECM environment of the tumor, thereby enabling further assessment of HAM penetration into deep tumor regions. As shown in Figure S6 (Supporting Information), the HAM+H_2_O_2_ group presented the highest FITC fluorescence intensity among all the groups. The results indicated that HAM with the motion ability could effectively penetrate the ECM barrier, further revealing the tumor penetration ability of HAM.

### 2.2 HAM‐RN Exhibits Strong Accumulation and Deep Penetration in Tumors

HAM‐RN was constructed by encapsulating HAM in a lipid layer composed of DPPC, cholesterol, and tumor‐targeting peptide DSPE‐PEG_2000_‐RGD nanoparticles (Figure 2J). DSPE‐PEG_2000_‐RGD nanoparticles were synthesized using a one‐step reaction and confirmed by ^1^H NMR (Figure S2C, Supporting Information). HAM‐RN were fabricated using a thin‐film dispersion technique: lipid formed into thin films under reduced pressure, then hydrated with HAM via ultrasound. The resulting HAM‐RN particles measured ≈134 ± 1.97 nm, with a polydispersity index of ≈0.253 ± 0.01, and a zeta potential of ≈6.58 ± 0.57 mV. TEM confirmed successful HAM encapsulation (Figure 2K). The storage stability, stability in cell culture medium, and stability in serum of HAM‐RN were assessed. As shown in Figure S7A–C (Supporting Information), no significant changes in size or PDI were observed for HAM‐RN stored at 4 °C over 12 days, demonstrating the great storage stability of HAM‐RN. No significant alterations in size or PDI occurred over 48 h in either RPMI‐1640 medium or 10% FBS solution, indicating that HAM‐RN maintained structural stability throughout systemic circulation following intravenous administration.

The release of HAM‐RN was further validated. As shown in Figure S7D (Supporting Information), the cumulative HAM release from the HAM‐RN group was significantly lower than the HAM‐RN+Laser group (*p* < 0.001). The cumulative release profiles of the same group were similar under different pH conditions, demonstrating that the DPPC‐containing HAM‐RN exhibited temperature‐sensitive release properties irrespective of the tumor microenvironment.

RGD peptides enable HAM‐RN to target tumor neovascularization in vivo. Using IR780 as a tracer, real‐time imaging showed IR780‐N (IR780‐RN without RGD) and IR780‐RN had significantly higher tumor fluorescence than free IR780 (IR780; Figure S8A, Supporting Information), with IR780‐RN demonstrating superior tumor accumulation due to RGD's tumor‐targeting properties. E*x vivo* fluorescence imaging confirmed that IR780‐N and IR780‐RN localized more effectively in tumors than IR780 alone (Figure S8B,C, Supporting Information), with IR780‐RN showing the highest accumulation. These findings demonstrate that RGD peptides enhance HAM‐RN targeting at tumor sites.

In vitro studies using Coumarin 6 (C6) as a tracer revealed greater uptake of C6‐RN by HUVECs compared to C6‐N (C6‐RN without RGD) after 0.5 h and 2 h incubation. Thus, the RGD peptide enabled effective targeting of HUVECs. These results were supported by flow cytometric analysis (Figure S9, Supporting Information).

HAM‐RN degrade under hyperthermia from laser‐irradiated HAM, releasing their contents (Figure S10, Supporting Information). Lipid encapsulation of HAM‐RN did not affect its ability to generate NO. Following 808 nm laser irradiation, HAM‐RN generated NO continuously for 10 h in H_2_O_2_ solution, with the NO generation curve not differing significantly from that of HAM (Figure 2L). The NO enhanced the penetration of HAM at tumor sites in vivo. Using Cy5.5‐NHS‐labeled HAM, immunofluorescence imaging revealed that, compared to HAM, HAM‐RN accumulated more at tumor sites. Moreover, HAM‐RN penetrated deeper into tumors, generating stronger fluorescence signals than non‐motile HM‐RN, which remained primarily at the tumor periphery (Figure 2M–O).

### 2.3 HAM‐RN Normalizes Tumor Blood Vessels via NO, Upregulating ZBTB46 to Promote Activated T‐Cell Infiltration Into Tumors Without Causing Metastasis

**Figure 3:**
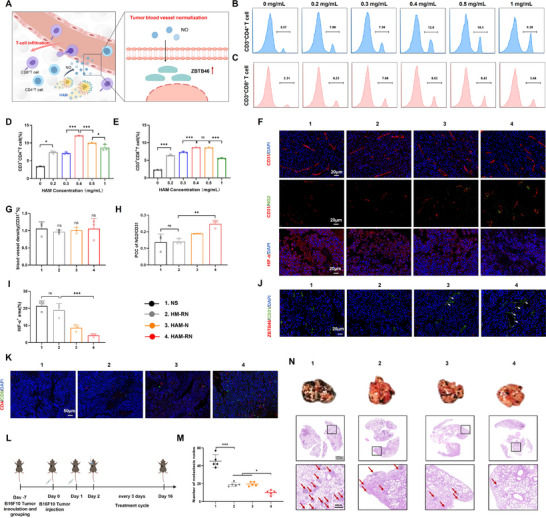
HAM‐RN normalizes tumor blood vessels via NO, upregulating ZBTB46 to promote activated T‐cell infiltration into tumors without causing metastasis. A), Schematic of NO promoting tumor blood vessel normalization by upregulating ZBTB46 to enhance T‐cell intratumoral infiltration. B–E), Proportion of CD3^+^CD4^+^ T and CD3^+^CD8^+^ T cells in tumors after treatment with different NO doses. F), Representative fluorescence micrographs; G–I) Analysis of (G) CD31^+^ blood vessel density (red), (H) NG2 pericyte (green) coverage, and (I) HIF‐α (red) staining, in tumor tissues of NS‐, HM‐RN‐, HAM‐N‐, and HAM‐RN‐treated mice; Scale bars: 20 µm. J), ZBTB46 expression in B16F10 tumors. Scale bar: 20 µm K), CD4^+^ T and CD8^+^ T‐cell infiltration in B16F10 tumors; Scale bar: 50 µm. L), Schedule of antimetastatic assessment in the B16F10 tumor pulmonary metastasis mouse model. M), Number of metastatic nodes in different groups. N), Representative pulmonary photographs and H&E‐stained sections; Scale bar: 2000, 400 µm. All the experiments were conducted with three independent replicates. Results are expressed as the mean ± SD. One‐way analysis of variance (ANOVA) with Tukey's post hoc test was used to assess significance. ^*^ *p* < 0.05, ^**^ *p* < 0.01, ^***^ *p* < 0.001, ns *p* > 0.05.

Promoting the normalization of tumor blood vessels through NO generated by HAM‐RN could improve the response to antitumor immunotherapy by increasing T ‐ cell infiltration (**Figure** **3**A). B16F10‐bearing mice were intravenously administered HAM‐RN (0, 0.2, 0.3, 0.4, 0.5, or 1 mg mL^−1^) to assess the dose‐dependent effects of NO on T‐cell infiltration. The infiltration of CD3^+^CD4^+^ (*p* < 0.001) and CD3^+^CD8^+^ (*p* < 0.001) T cells was significantly lower in the HAM‐RN‐treated group than in the 0.5 mg mL^−1^ HAM‐RN group, indicating that low doses of NO promoted tumor blood vessel normalization and T‐cell infiltration. Compared with 0.3 mg mL^−1^ HAM‐RN, 0.4 mg mL^−1^ HAM‐RN significantly promoted CD3^+^CD4^+^ (*p* < 0.001) and CD3^+^CD8^+^ (*p* < 0.001) T‐cell infiltration into B16F10 tumors. Compared with 0.5 mg mL^−1^ HAM‐RN, 0.4 mg mL^−1^ HAM‐RN increased CD3^+^CD4^+^ T‐cell infiltration (*p* < 0.001). However, no significant differences were observed in CD3^+^CD8^+^ T‐cell infiltration in tumors treated with 0.5 or 0.4 mg mL^−1^ HAM‐RN (Figure 3B–E). Hence, 0.4 mg mL^−1^ HAM‐RN was the most suitable dose for enhancing T‐cell infiltration.

After treatment with 0.4 mg mL^−1^ HAM‐RN under laser irradiation, tumors were resected for analysis. Immunohistochemical staining of CD31 (vessel marker), NG2 (pericyte‐specific marker), and hypoxia‐inducible factor‐α (HIF‐α; Figure 3F–I) revealed that, compared to NS (*p* < 0.001), HM‐RN+L (*p* < 0.001), and HAM‐N+L (*p* < 0.001), HAM‐RN+L did not significantly increase the blood vessel density. Quantitative analysis of the pericyte coverage ratio (NG2/CD31) revealed that the HAM‐RN+L group had the highest pericyte coverage, suggesting greater tumor blood vessel maturity. HAM‐RN+L treatment also significantly reduced the HIF‐α‐positive area in tumor sections, suggesting improved hypoxia, while the effects of HM‐RN+L treatment did not differ significantly from those of NS treatment. These results suggest that the NO produced by HAM‐RN promotes the normalization of tumor blood vessels.

Higher ZBTB46 expression in endothelial cells reportedly promotes tumor blood vessel normalization.^[^ ^26^ ^]^ Thus, the association between the blood vessel normalization effects of NO and ZBTB46 expression at the tumor site was evaluated. The HAM‐RN group showed the highest co‐localization of ZBTB46 and CD31, suggesting that NO promoted tumor blood vessel normalization by upregulating ZBTB46 expression in endothelial cells (Figure 3J). Moreover, CD4 and CD8 staining revealed increased CD4^+^ and CD8^+^ T‐cell infiltration of tumor tissue sections within the HAM‐RN group compared to the NS group (Figure 3K), suggesting that the NO produced by HAM‐RN+L also promotes T‐cell infiltration via normalized blood vessels.

Additionally, normalizing tumor blood vessels improved formulation accumulation in tumors and potentiated the photothermal therapeutic efficiency mediated by HAM‐RN. The photothermal conversion capacity of HAM‐RN was quantitatively assessed in vivo 12 h after administration. B16F10 tumor‐bearing mice treated with NS, HAM, or HAM‐RN plus laser irradiation showed tumor temperature increases of 4.8 ± 0.14, 10.5 ± 0.92, and 12.8 ± 0.37 °C, respectively. This further confirmed enhanced photothermal effects alongside tumor vessel normalization (Figure S11, Supporting Information).

To determine whether normalizing tumor blood vessels with NO induces tumor metastasis while promoting T‐cell infiltration, a mouse model of pulmonary metastasis was established by intravenously injecting tumor cells (2 × 10^6^ cells/mouse) into B16F10 tumor‐bearing C57BL/6J mice (Figure 3L). After 16 days of treatment, the lungs were harvested, and pulmonary metastasis was evaluated. The HAM‐RN+L group had significantly fewer metastatic nodes than the other groups, indicating reduced metastasis risk. Representative lung photographs and histopathological H&E‐stained sections from the different groups showed the antimetastatic effects of HAM‐RN+L (Figure 3M,N).

### 2.4 HAM‐RN Generates Whole‐Tumor Antigens via ICD Under Laser Irradiation and Captures Antigens to Promote DC Maturation

**Figure 4:**
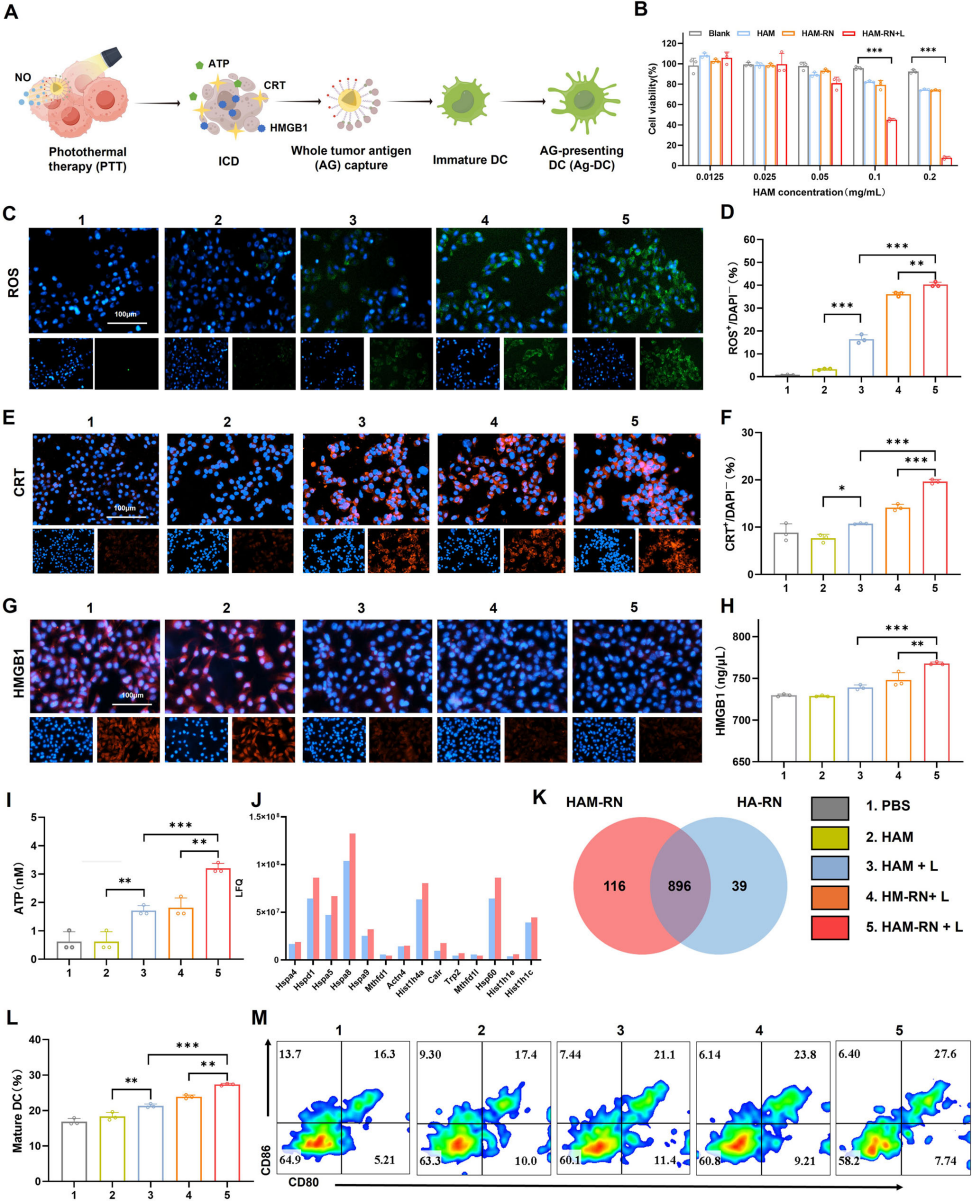
HAM‐RN generates whole‐tumor antigens via ICD under laser irradiation and captures antigens to promote DC maturation. A), Schematic of dying tumor cells promoting DC maturation through ICD and whole‐tumor antigen capture. B), B16F10 cell viabilities in different groups: unloaded nanoparticles (Blank), HAM, HAM‐RN, and HAM‐RN+Laser (HAM‐RN+L). C), Fluorescence microscopy images of B16F10 cells stained with DCFH‐DA from different groups. D), Flow cytometric quantification of ROS generation in B16F10 cells. E,F), Fluorescence images and flow cytometric quantification of CRT exposure in B16F10 cells. G), Fluorescence images of HMGB1 in B16F10 cells. H), Quantitative analysis of HMGB1 release. I), Quantification of ATP secretion. J), Relative abundance of B16F10‐specific tumor antigens captured by HAM‐RN and HA‐RN after laser irradiation. K), Number of B16F10 cell protein types captured by HAM‐RN and HA‐RN after laser irradiation. L,M), Flow cytometric results of mature DCs after treatment. All experiments were conducted with three independent replicates. Results are expressed as the mean ± SD. One‐way analysis of variance (ANOVA) with Tukey's post hoc test was used to assess significance. ^*^ *p* < 0.05, ^**^ *p* < 0.01, ^***^ *p* < 0.001.

Following laser irradiation, HAM‐RNs generate ROS and trigger ICD, which promotes the release of damage‐associated molecular patterns (DAMPs), including cell membrane calreticulin (CRT), secreted high‐mobility group box 1 (HMGB1), and ATP. Meanwhile, whole‐tumor antigens promote DC maturation (**Figure** **4**A).

HAM‐RN's cytotoxicity was assessed using a methyl thiazolyl tetrazolium (MTT) assay. Unloaded nanoparticles (blank), free HAM (HAM), and HAM‐RN were nontoxic to B16F10 cells without laser irradiation. However, exposure to 808 nm irradiation induced notable toxic effects on B16F10 cells in the HAM‐RN group, suggesting that HAM‐RN is nontoxic in the absence of laser irradiation but induces tumor cell killing under irradiation. (Figure 4B).

The generation of ROS in B16F10 cells was subsequently assessed. The NS and HAM‐RN groups without laser irradiation did not generate ROS. Laser irradiation led to ROS production in B16F10 cells treated with HAM, HM‐RN, or HAM‐RN, with the HAM‐RN+L exhibiting the strongest effect (Figure 4C,D).

CRT, HMGB1, and ATP expression levels in B16F10 cells were measured as indicators of ICD. The fluorescence signals of CRT in the NS and HAM‐RN groups without laser irradiation increased slightly; however, higher levels were observed in the HAM‐RN+L group compared with the HAM+L and HM‐RN+L groups (Figure 4E,F). Additionally, compared with the HAM+L and HM‐RN+L groups, nuclear HMGB1 fluorescence was lower in the HAM‐RN+L group (Figure 4G). The culture supernatants of the HAM+L, HM‐RN+L, and HAM‐RN+L groups also exhibited high HMGB1 and ATP levels, with the highest levels detected in the HAM‐RN+L group (Figure 4H,I). These results suggest that combining HAM‐RN with irradiation may be an effective strategy for inducing ICD.

The ICD effect can enhance whole‐tumor antigen generation, while exposed antigens can be captured by Mal on the surface of HAM. To investigate the antigen capture ability of HAM‐RN, the antigens from B16F10 cell lysates that were captured by HAM‐RN and HA‐RN (HAM‐RN without Mal modification) were identified. HAM‐RN captured a broader range of antigens than HA‐RN, with greater intensity of adsorbed tumor antigens, including DAMPs and tumor neoantigens (Figure 4J,K). This demonstrated that the HAM‐RN+L group had a more extensive antigen capture capacity that was not impacted by the lipid nanoparticle coating.

The transwell system was used to assess the capacity of HAM‐RN to accelerate DC maturation in vitro. The highest proportion of DC maturation was observed in the HAM‐RN+L group, with an increased frequency of 27.6% (Figure 4L,M). Thus, HAM‐RN+L treatment potently induces ICD to release whole‐tumor antigens and promote DC maturation, thereby activating T cells.

### 2.5 VEGFC‐RN Reconstructs Tumor‐Associated Lymphatic Vessels to Increase Lymphatic Drainage and Promote DC Accumulation in TDLNs

**Figure 5:**
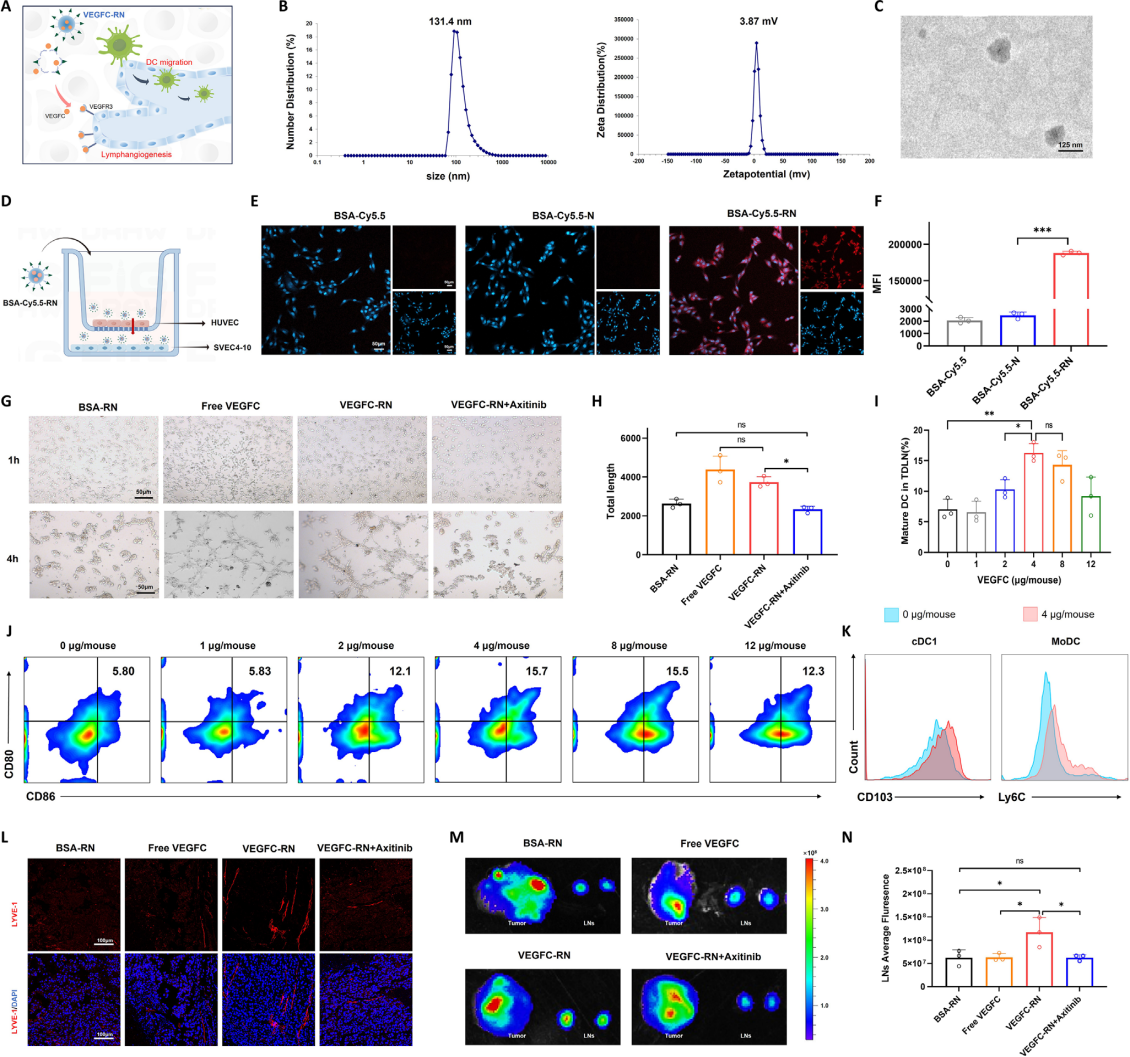
VEGFC‐RN reconstructs tumor‐associated lymphatic vessels to increase lymphatic drainage and promote DC accumulation in TDLNs. A), Schematic of the acting mechanism of VEGFC‐RN to promote lymphangiogenesis. B), Particle size and zeta potential of VEGFC‐RN. C), Transmission electron microscopy image of VEGFC‐RN. D), Schematic diagram of the transwell assay. E), Representative images of the transcellular transport of BSA‐Cy5.5, BSA‐Cy5.5‐N, and BSA‐Cy5.5‐RN through HUVECs subjected to vascular mimicry in the transwell system; Scale bar: 50 µm. F), Fluorescence intensity of the transcellular transport of BSA‐Cy5.5, BSA‐Cy5.5‐N, and BSA‐Cy5.5‐RN through HUVEC vascular mimicry simulated with the transwell system, quantified by flow cytometry. G), Representative images of the in vitro tube formation assay after treatment with BSA‐RN, free VEGFC, VEGFC‐RN, or VEGFC‐RN+Axitinib; Scale bar: 50 µm. H), Total lengths of tubes formed after treatment with BSA‐RN, free VEGFC, VEGFC‐RN, or VEGFC‐RN+Axitinib. I,J), Flow cytometric analysis of mature DCs (CD11c^+^CD80^+^CD86^+^ cells) after treatment with different doses of VEGFC within TDLNs. K), Flow cytometric analysis of cDC1s (CD11c^+^CD103^+^ cells) and MoDCs (CD11c^+^Ly6C^+^ cells). L), Representative fluorescence micrographs of B16F10 tumor sections after LYVE‐1 staining; Scale bar: 100 µm. M), *Ex vivo* imaging of tumors and LNs after treatment with HAM‐RN combined with BSA‐RN, free VEGFC, VEGFC‐RN, or VEGFC‐RN+Axitinib. N), Average fluorescence intensities of LNs after treatment with HAM‐RN combined with BSA‐RN, free VEGFC, VEGFC‐RN, or VEGFC‐RN+Axitinib. All experiments were conducted with three independent replicates. Results are expressed as the mean ± SD. One‐way analysis of variance (ANOVA) with Tukey's post hoc test was used to assess significance. ^*^ *p* < 0.05, ^**^ *p* < 0.01, ^***^ *p* < 0.001, ns *p* > 0.05.

VEGFC‐RN exhibits temperature‐responsive degradation, releasing VEGFC at tumor sites to activate VEGFR3, which promotes lymphangiogenesis and facilitates antigen‐presenting DC migration to TDLNs (**Figure** **5**A). VEGFC‐RN measured ≈128 ± 2.39 nm in diameter, with a polydispersity index of 0.205 ± 0.01 and a zeta potential near 4.41 ± 0.38 mV (Figure 5B). As shown in the transmission electron microscope image, VEGFC‐RN was spherical in shape and uniform (Figure 5C).

The mediation of VEGFC‐RN transcellular transport by RGD peptides was assessed using a transwell system with HUVECs cultured in the upper chamber and murine lymphatic endothelial cells (SVEC4‐10) in the bottom. BSA‐Cy5.5 was used in place of VECFC to observe the transcellular transport of VEGFC‐RN (Figure 5D). A significant Cy5.5 fluorescence signal could be observed at the bottom of the transwell system in the BSA‐Cy5.5‐RN group, which could not be observed in the free BSA‐Cy5.5 (BSA‐Cy5.5) or BSA‐Cy5.5‐RN without RGD modification (BSA‐Cy5.5 ‐ N) groups, revealing that VEGFC‐RN could cross blood vessels via RGD peptides and exhibit increased uptake by lymphatic endothelial cells (Figure 5E). The fluorescence intensities quantified with flow cytometry also verified this finding (Figure 5F).

Analysis of tube formation in SVEC4‐10 cells confirmed the capacity of VEGFC‐RN to promote tumor lymphangiogenesis in vitro (Figure 5G). As shown in the transmitted light images, no branches were observed at 1 h, yet at 4 h, the BSA‐RN group presented fewer branches than the free VEGFC and VEGFC‐RN groups. Meanwhile, branch formation was inhibited by VEGFC‐RN and Axitinib—small‐molecule inhibitors of the VEGFR3 receptor. Quantitative assessment of tube length further revealed that treatment with free VEGFC or VEGFC‐RN restored the tube formation capacity of SVEC4‐10 cells (Figure 5H). Collectively, these results demonstrate that VEGFC facilitates tumor lymphangiogenesis by binding VEGFR3 on lymphatic endothelial cells.

The concentration of VEGFC was selected based on the proportion of mature DCs within the TDLNs in the B16F10‐bearing mouse model. Mice received HAM‐RN (0.4 mg mL^−1^, 0.1 mL) and varying doses of VEGFC‐RN (0, 1, 2, 4, 8, or 12 µg mouse^−1^) every three days. Two days after administration, the mice were subjected to 808 nm laser irradiation (1.2 W cm^−2^, 5 min), and TDLNs were harvested for DC isolation after 16 days. The proportion of mature DCs (CD11c^+^CD80^+^CD86^+^ cells) in the 4 µg mouse^−1^ VEGFC group was significantly higher than in the 0, 1, and 2 µg mouse^−1^ groups, with no significant difference observed between the 4 and 8 µg mouse^−1^ groups. Accordingly, 4 µg mouse^−1^ was selected as the optimal therapeutic dose for in vivo VEGFC application (Figure 5I,J).

Conventional type 1 dendritic cells (cDC1s) and monocyte‐derived dendritic cells (MoDCs) are critical in CD8^+^ T cell priming.^[^ ^27^, ^28^ ^]^ Thus, subsequent analysis assessed DC subtypes within TDLNs post‐treatment with HAM‐RN and VEGFC‐RN. The proportions of cDC1s (CD11c^+^CD103^+^ cells) and MoDCs (CD11c^+^Ly6C^+^ cells) were higher in the 4 µg mouse^−1^ group relative to the 0 µg mouse^−1^ group (Figure 5K). The accumulation of cDC1s and MoDCs in TDLNs substantiates the conclusion that reconstructed tumor‐associated lymphatic vessels improve the T‐cell response and enhance antitumor effects.

Finally, the ability of VEGFC‐RN to increase lymphatic drainage in vivo was assessed by injecting B16F10‐bearing mice with BSA‐RN, free VEGFC, VEGFC‐RN, or VEGFC‐RN combined with axitinib (VEGFC‐RN+Axitinib). After two days, HAM‐RN (with Cy5.5 instead of Mal) was administered, followed by 808 nm laser irradiation. LYVE‐1 staining of the tumor's lymphatic vessels revealed increased intratumoral lymphatic vessels in the VEGFC‐RN group (Figure 5L). NIFR imaging and quantitative analyses further showed that while Cy5.5 fluorescence at the tumor site did not differ significantly among the different groups, lymph node fluorescence was higher in the VEGFC‐RN group compared to the others (Figure 5M,N). These results demonstrate that VEGFC‐RN promotes tumor lymphangiogenesis and TDLN HAM accumulation by enhancing lymphatic drainage, supporting improved antigen utilization.

### 2.6 HAM‐RN Combined with VEGFC‐RN Reconstructs Tumor‐Associated Blood and Lymphatic Vessels and Promotes T‐Cell Immune Memory in the B16F10 Primary Tumor Mouse Model

**Figure 6:**
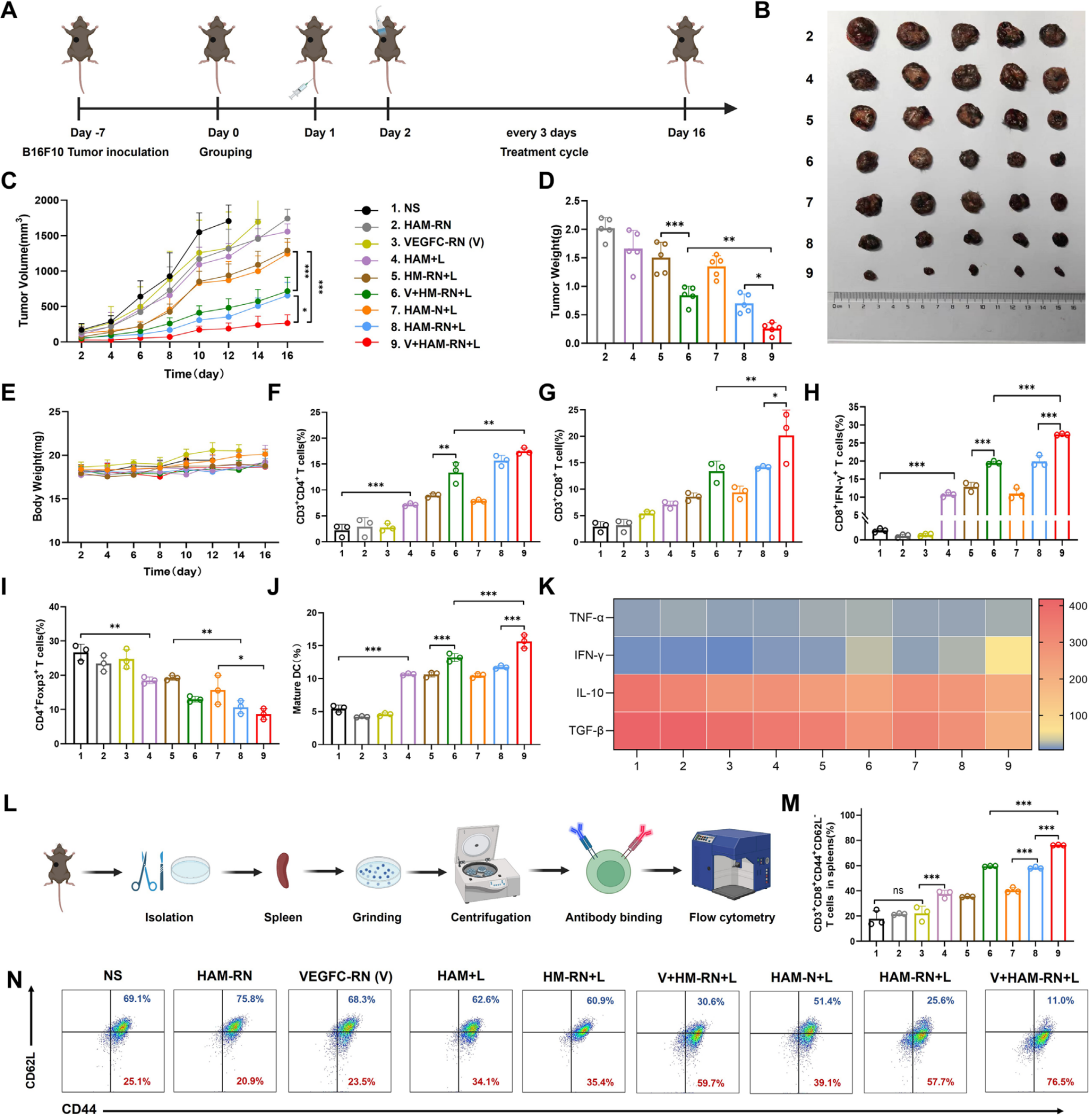
HAM‐RN combined with VEGFC‐RN reconstructs tumor‐associated blood and lymphatic vessels and promotes T‐cell immune memory in the B16F10 primary tumor mouse model. A), Regimen for the B16F10 tumor mouse model with different formulations. B), Photographs of tumors, C), tumor volume curves, D), tumor weights, and E), body weights in different groups at the 16‐day endpoint (*n* = 5 biologically independent animals per group). Flow cytometric quantification of F), intratumoral CD4^+^ T and G), CD8^+^ T cells, H), CTLs, I), Tregs, and J), mature DCs in the TDLNs. K), Heatmap of the intratumoral levels of TNF‐α, IFN‐γ, IL‐10, and TGF‐β in the B16F10 tumor. L), Schematic for T_CM_ and T_EM_ detection, M,N), Flow cytometric quantification of T_CM_ and T_EM_ in splenic tissues. All experiments were conducted with three independent replicates. Results are expressed as the mean ± SD. One‐way analysis of variance (ANOVA) with Tukey's post hoc test was used to assess significance. ^*^ *p* < 0.05, ^**^ *p* < 0.01, ^***^ *p* < 0.001.

The in vivo antitumor effects of HAM‐RN and VEGFC‐RN were assessed in female C57BL/6J mice bearing B16F10 tumors across nine experimental groups: normal saline (NS), HAM‐RN, VEGFC‐RN (V), free HAM+laser (HAM+L), HM‐RN+laser (HM‐RN+L), VEGFC‐RN+HM‐RN+laser (V+HM‐RN+L), HAM‐N+laser (HAM‐N+L), HAM‐RN+laser (HAM‐RN+L), and VEGFC‐RN+HAM‐RN+laser (V+HAM‐RN+L). Different formulations were injected through the tail vein, and the HAM+L, HM‐RN+L, V+HM‐RN+L, HAM‐N+L, HAM‐RN+L, and V+HAM‐RN+L groups were subjected to 808 nm laser irradiation once for 5 min at 1.2 W cm^−2^. Treatment cycles were administered at 3‐day intervals over a 16‐day period, and tumor volumes and body weights were recorded every two days (**Figure** **6**A). The final average tumor weight in mice treated with V+HAM‐RN+L was the lowest (Figure 6B). The tumor volumes in the NS and V groups reached ≈2000 mm^3^ after 12 and 14 days of treatment, respectively. HAM‐RN+L and V+HAM‐RN+L effectively inhibited tumor growth, and the tumor volume decreased significantly after treatment with V+HAM‐RN+L, indicating effective tumor inhibition by simultaneous reconstruction of tumor‐associated blood and lymphatic vessels (Figure 6C; Figure S12, Supporting Information). After 16 days of treatment, the tumor volumes were similar (Figure 6D). Apoptosis and necrosis were evaluated by Ki67 and TUNEL staining of the tumor tissue sections (Figure S13, Supporting Information), revealing fewer Ki67‐positive tumor cells and more TUNEL‐stained cells in the V+HAM‐RN+L group, indicating low levels of tumor cell proliferation and enhanced apoptosis.

Furthermore, the body weights of mice treated with different formulations did not differ significantly between groups (Figure 6E). Histopathological evaluation of H&E‐stained organ tissues (heart, liver, spleen, lungs, and kidneys) revealed no significant pathological alterations in any group (Figure S14A, Supporting Information). The key biomarkers, such as ALT, AST, and IL‐6 in the serum of mice were assessed. As shown in Figure S15 (Supporting Information), no statistically significant differences of the AST, ALT, and IL‐6 levels were observed in different groups, and all the values remained within physiological reference ranges. These findings collectively indicate that sustained immune activation mediated by the combined HAM‐RN and VEGFC‐RN therapeutic regimen, alongside cumulative nanomotor activity, did not induce systemic inflammation. The treatment demonstrated favorable preliminarsafety in mice.

Modulation of the immune phenotype within the TME was also evaluated by assessing immune cell populations—including CD3^+^CD4^+^ T, CD3^+^CD8^+^ T cells, CTLs (CD8^+^IFN‐γ^+^ T cells), and Tregs (CD4^+^Foxp3^+^ T cells)—as well as cytokine concentrations (TNF‐α, IFN‐γ, IL‐10, and TGF‐β) in tumors, and the proportion of mature DCs within TDLNs. Quantitative analysis revealed significantly increased infiltration levels of CD3^+^CD4^+^ T and CD3^+^CD8^+^ T cells in the V+HAM‐RN+L group, with 7.86‐fold and 6.88‐fold increases, respectively, compared to the NS group, suggesting that V+HAM‐RN+L effectively promotes intratumoral T‐cell infiltration. Relative to the HAM‐RN‐L group, the V+HM‐RN‐L group exhibited a marked increase in the proportion of CD3^+^CD4^+^ T cells (*p* < 0.001); similarly, CD3^+^CD8^+^ T‐cell proportions in the V+HAM‐RN‐L group increased from 14.2 ± 0.21% to 20.2 ± 3.93%, indicating that the tumor‐associated lymphatic vessel reprogramming significantly enhanced T‐cell activation. Treatment with V+HAM‐RN‐L resulted in further increases in CD3^+^CD4^+^ and CD3^+^CD8^+^ T cells (*p* < 0.001 for both), highlighting that simultaneous reconstruction of tumor‐associated blood and lymphatic vessels can effectively enhance T‐cell activation and intratumoral infiltration (Figure 6F,G; Figure S16A, Supporting Information).

The proportion of infiltrating CTLs increased to 27.37 ± 0.17% after treatment with V+HAM‐RN+L, exceeding the V+HM‐RN+L group (19.5 ± 0.37%, *p* < 0.001), indicating improved CTL penetration due to tumor blood vessel normalization (Figure 6H; Figure S16B, Supporting Information). Meanwhile, the proportion of Tregs was the lowest in the V+HAM‐RN+L group, suggesting reversal of immunosuppressive effect within the TME following treatment (Figure 6I; Figure S16C, Supporting Information). Moreover, among all tested formulations, V+HAM‐RN+L exhibited the strongest ability to promote DC maturation. The V+HM‐RN+L group had a significantly higher proportion of mature DCs than the HM‐RN+L group (*p* < 0.001), demonstrating the positive impact of lymphatic vessel reprogramming on mature DC migration to TDLNs. Additionally, simultaneous reprogramming of blood and lymphatic vessels via V+HAM‐RN+L treatment further increased mature DC counts compared to the V+HM+RN+L group (*p* < 0.001), reflecting a strengthened immune response (Figure 6J; Figure S16D, Supporting Information).

In order to exclude the independent effects of laser‐induced local heating to immune and cellular responses, the cytotoxicity of laser via the MTT assay in vitro was assessed (Figure S17A, Supporting Information). The 808 nm laser irradiation showed no toxicity to B16F10 cells. Furthermore, the populations of T cells within tumors and mature DCs within TDLNs of mice subjected to laser irradiation were quantified. As shown in Figure S17B,C (Supporting Information), the proportions of CD3^+^CD4^+^T, CD3^+^CD8^+^T cells, and mature DCs in the Laser group showed no significant difference compared to the NS group, indicating that laser‐induced local heating could not independently affect immune and cellular responses.

Cytokine analysis revealed elevated TNF‐α and IFN‐γ levels alongside reduced IL‐10 and TGF‐β in the V+HAM‐RN+L group (Figure 6K; Figure S18A, Supporting Information). Overall, alterations in immune cell populations and cytokine profiles confirmed that V+HAM‐RN+L treatment markedly enhanced immune cell infiltration and activation—specifically DCs, T cells, and CTLs—reduced Treg proportions, and modulated cytokine release through reprogramming tumor‐associated blood and lymphatic vessels to reverse the suppressive TME.

Activation of memory T cells following primary tumor therapy contributes to sustained immune memory. Accordingly, central memory T cells (T_CM_, CD3^+^CD8^+^CD44^+^CD62L^+^) and effector memory T cells (T_EM_, CD3^+^CD8^+^CD44^+^CD62L^−^) were isolated from the spleen, labeled, and analyzed to assess antitumor effects (Figure 6L). Compared with the NS group, the proportion of T_EM_ in the V + HAM‐RN+L group increased significantly from 25.1% to 76.5%, while that of T_CM_ decreased from 69.1% to 11.0%, indicating that the V+HAM‐RN+L regimen primarily relies on T_EM_ to establish long‐term immune memory (Figure 6M,N).

### 2.7 HAM‐RN Combined with VEGFC‐RN Strongly Initiates and Maintains the T‐Cell Immune Response to Inhibit Tumor Progression in the 4T1 Primary Tumor Mouse Model

**Figure 7:**
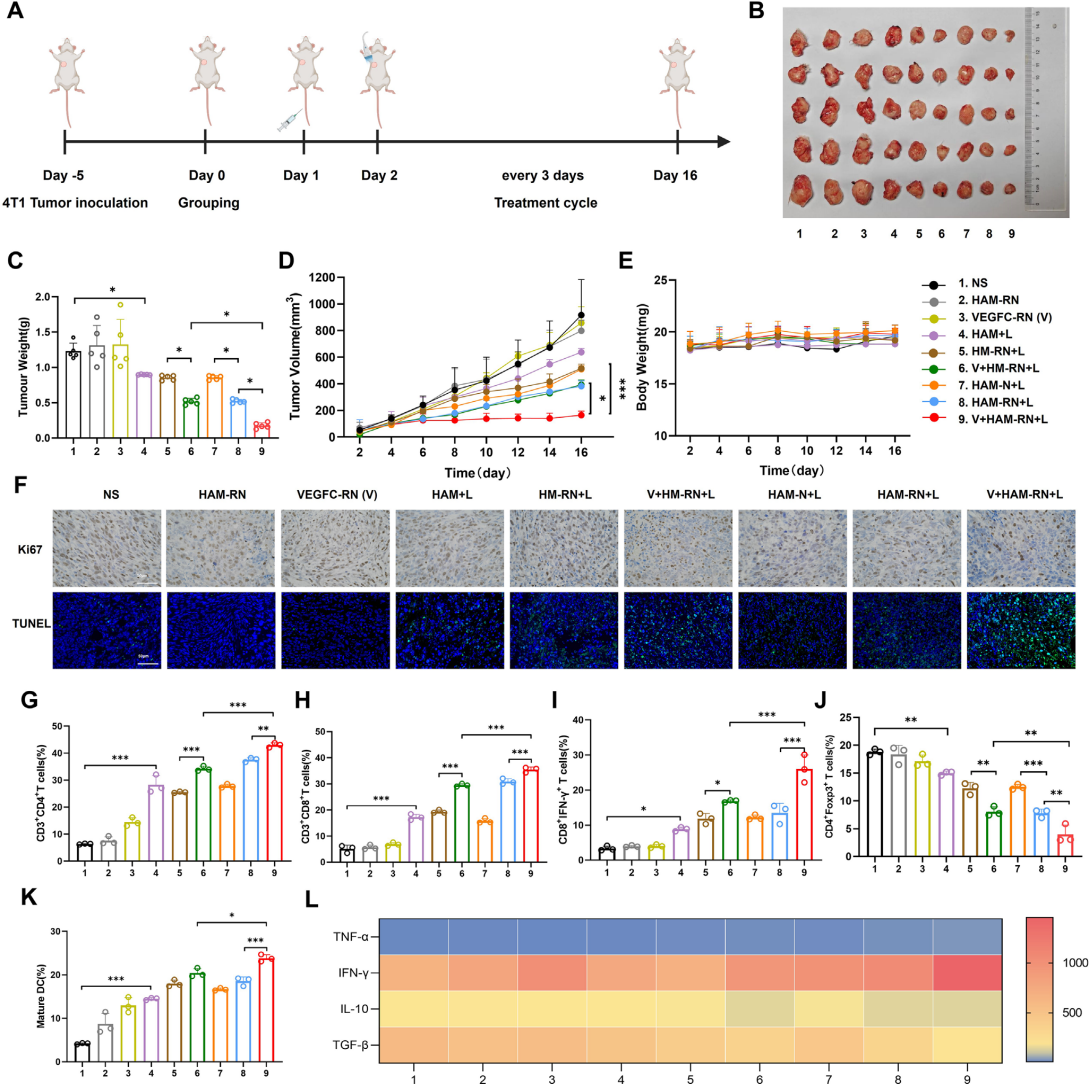
HAM‐RN combined with VEGFC‐RN strongly initiates and maintains the T‐cell immune response to inhibit tumor progression in the 4T1 primary tumor mouse model. A), Treatment regimens for the 4T1 tumor mouse model. B), Photographs of tumors, C), average tumor weights, D), average tumor growth curves, and E), body weights of 4T1‐bearing mice receiving different treatments at the treatment endpoint *(n* = 5 biologically independent animals per group). F), Ki67 and TUNEL staining of tumor tissues from the 4T1 primary tumor; Scale bar: 50 µm. Flow cytometric quantification of G), CD3^+^CD4^+^ T cells, H), CD3^+^CD8^+^ T cells, I), CTLs (CD8^+^IFN‐γ^+^ T cells), and J), Tregs (CD4^+^Foxp3^+^ T cells) in tumors and K), mature DCs within the TDLNs. L), Quantitative ELISA analysis of the intratumoral cytokine levels at the study endpoint. All experiments were conducted with three independent replicates. Results are expressed as the mean ± SD. One‐way analysis of variance (ANOVA) with Tukey's post hoc test was used to assess significance. ^*^ *p* < 0.05, ^**^ *p* < 0.01, ^***^ *p* < 0.001.

The antitumor effects of reconstructing the tumor‐associated blood and lymphatic vessels were assessed in a 4T1 primary mouse model. Each mouse was inoculated with 4T1 cells in the upper left mammary gland and treated for 5 days (**Figure** **7**A). The 4T1 tumor‐bearing mice were grouped in the same way as the B16F10 tumor mouse model. The tumor volumes of the different groups were measured every 2 days during the 16‐day treatment, and the tumors were removed and weighed after treatment. As shown by the tumor photographs, average tumor weights, and tumor growth curves (Figure 7B–D), the mice in the V+HAM‐RN+L group presented the slowest tumor growth trend and had smaller tumor volumes and lighter tumor weights than those in the V+HM‐RN+L group, demonstrating that blood vessel normalization was effective for antitumor therapy. Compared with those in the HM‐RN+L group, the V+HM‐RN+L group presented smaller tumor volumes and lighter tumor weights, indicating that lymphatic vessel reprogramming mediated by VEGFC‐RN was crucial for antitumor therapy. Meanwhile, body weights were similar among the groups (Figure 7E), and histological analysis of the major organs (heart, liver, spleen, lungs, and kidneys) revealed no significant toxicity (Figure S14B, Supporting Information), suggesting that V+HAM‐RN+L was safe. Ki67 and TUNEL staining further indicated less tumor cell proliferation and more apoptosis in the V+HAM‐RN+L group (Figure 7F).

Changes in immune cell proportions were also evaluated in the TME, revealing CD3^+^CD4^+^ T‐cell, CD3^+^CD8^+^ T‐cell, and CTL populations in the V+HM‐RN‐L group (34.2 ± 0.77%, 29.5 ± 0.45%, and 16.9 ± 0.26%, respectively), which were 1.34‐, 1.53‐, and 1.43‐fold larger than in the HM‐RN‐L group, indicating effective T‐cell activation through lymphatic vessel reprogramming. The proportions of CD3^+^CD4^+^ T cells, CD3^+^CD8^+^ T cells, and CTLs in the V+HAM‐RN‐L group increased by 1.26‐fold (43 ± 0.70%), 1.20‐fold (35.4 ± 0.82%), and 1.55‐fold (26.1 ± 3.2%) compared to the V+HM‐RN‐L group, suggesting that simultaneous lymphatic and blood vessel reconstruction further promoted T‐cell activation and intratumoral infiltration (Figure 7G–I). The proportion of Tregs was smallest in the V+HAM‐RN+L group, demonstrating reversal of the immunosuppressive TME (3.97 ± 1.37%; Figure 7J). Additionally, the mature DC population in the V+HAM‐RN+L group (23.8 ± 0.66%) was 1.17‐fold and 1.29‐fold larger than in the V+HM‐RN+L and HAM‐RN+L groups, respectively (Figure 7K), supporting improved DC maturation. Finally, cytokine analysis revealed higher levels of TNF‐α and IFN‐γ, and lower levels of IL‐10 and TGF‐β in the V+HAM‐RN+L group (Figure 7L; Figure S18B, Supporting Information).

Overall, combined HAM‐RN and VEGFC‐RN enhanced immune‐promoting cell infiltration and cytokine release while inhibiting immunosuppressive cells and cytokines, resulting in more robust antitumor immune effects in the 4T1 mouse model.

## 3 Conclusion

Insufficient intratumoral infiltration of immune cells leads to poor efficiency or treatment failure in antitumor immunotherapy. Herein, a multifunctional NO‐driven Janus nanomotor (HAM) was designed as a novel in situ cancer vaccine that normalizes tumor blood vessels and enhances T‐cell infiltration and antitumor effects. HAM produces ROS under laser irradiation, inducing ICD and increasing whole‐tumor antigen production; these antigens are then captured to boost DC‐mediated presentation. The resulting ROS reacts with surface‐modified Arg, generating NO, which drives tumor penetration and blood vessel normalization, thereby supporting T‐cell infiltration. Combining HAM with VEGFC facilitates reconstruction of tumor‐associated lymphatic vessels, improving DC migration to TDLNs for optimal T‐cell activation. This approach not only improves antigen presentation by DCs and T‐cell activation but also underscores the importance of blood and lymphatic vessels in sustaining the cancer‐immune cycle and enabling T‐cell infiltration.

In this study, NO was selected to normalize tumor blood vessels, simulating the effects of antiangiogenic therapy. Unlike traditional NO donors, the NO‐driven Janus nanomotors overcome the limited penetration caused by interstitial fluid pressure within solid tumors, allowing them to function as an in situ vaccine by inducing tumor blood vessel normalization in deep tumor regions and promoting activated T‐cell infiltration. Additionally, this strategy increases ZBTB46 expression in endothelial cells at tumor sites via NO, further supporting vessel normalization and T‐cell infiltration without promoting tumor growth. In future research, these NO‐driven Janus nanomotors can be combined with T‐cell‐based immunotherapies, such as immune checkpoint inhibitors or CAR‐T cells, to overcome current limitations in T‐cell infiltration.

## 4 Experimental Section

### Materials

1,2‐Dipalmitoyl‐sn‐glycero‐3‐phosphocholine (DPPC) was procured from AVT (Shanghai) Pharmaceutical Tech Co., Ltd (Shanghai, China). Cholesterol, L‐Arginine, Hydroxylammonium chloride, SATA, Coumarin 6, and IR780 were purchased from Shanghai Aladdin Bio‐Chem Technology Co., Ltd (Shanghai, China). Trisodium citrate dihydrate was procured from Sinopharm Chemical Reagent Co., Ltd (Shanghai, China). NH_2_‐PEG_2000_‐Mal and NH_2_‐PEG_2000_‐NH_2_ were purchased from Shanghai Ponsure Biotech, Inc. (Shanghai, China). DSPE‐PEG_2000_‐NHS was obtained from Xi'an RuiXi Biological Technology Co., Ltd (Xi'an, China). RGD was purchased from Leon Biological Technology Co., Ltd. (Nanjing, China). HMGB1 ELISA kits, Nitric Oxide(NO)Content Assay Kit, and methylthiazol tetrazolium (MTT) were acquired from Beijing Solarbio Science & Technology Co., Ltd (Beijing, China). Mouse Granulocyte‐Macrophage Colony‐Stimulating Factor (GM‐CSF), and Mouse IL‐4 were obtained from Novoprotein (Shanghai, China). Mouse Vascular Endothelial Growth Factor C (VEGFC) was purchased from Sino Biological Inc. (Beijing, China). Axitinib was purchased from Shandong Sparkjade Biotechnology Co., Ltd (Shandong, China). Matrigel was obtained from Corning Inc. (United States). Anti‐mouse CD3, CD4, CD8, IFN‐γ, CD11c, CD80, CD86, Foxp3, CD44, and CD62L antibodies were obtained from Biolegend (San Diego, United States).

### Cells

Murine melanoma cell line (B16F10), Mouse breast cancer cells (4T1), Human umbilical vein endothelial cell line (HUVEC) were purchased from the Chinese Academy of Sciences (China, Shanghai Institutes for Biological Sciences). Mouse bone marrow‐derived dendritic cell line (DC2.4) was procured from Hunan Fenghui Biotechnology Co., Ltd. (Hunan, China), while murine lymphatic endothelial cell line (SVEC4‐10) was acquired from Pricella Life Science & Technology Co., Ltd (Wuhan, China). Bone marrow‐derived dendritic cells (BMDCs) were isolated from female C57BL/6J mouse femurs. B16F10 and 4T1 cell lines were maintained in RPMI 1640 supplemented with penicillin/streptomycin (1%) and fetal bovine serum (10%). DC2.4 cells were cultured in DMEM containing identical antibiotic and serum concentrations. Both HUVEC and SVEC4‐10 cell lines were cultured in DMEM/F‐12 with equivalent supplementation. BMDCs were cultured in RPMI 1640 with 20 ng mL^−1^ each of GM‐CSF and IL‐4, with half‐medium replacement every 2 days prior to experimental use at day 7. All the cells were cultured in 37 °C and 5% CO_2_, and showed negative for mycoplasma contamination.

### Animals

6–8 week’ female C57BL/6J and BALB/c mice were provided by SPF Biotechnology Co., Ltd. (Beijing, China). The tumor‐bearing mice were euthanized with carbon dioxide when tumor volumes reached ≈2000 mm^3^. All animal studies were conducted in compliance with the guidelines established by the Animal Experiment Ethics Review Board of Shandong University (Approval number 21 083).

### Synthesis of SATA‐PEG_2000_‐Mal

The reaction between SATA and NH_2_‐PEG_2000_‐Mal was performed at a ratio of 1:1.2 (mol/mol). After 16‐h magnetic stirring, the crude product underwent aqueous dialysis (48 h) followed by lyophilization to yield the target compound SATA‐PEG2000‐Mal. Structural confirmation of NH_2_‐PEG_2000_‐Mal was achieved through ^1^H‐NMR analysis (600 MHz, DMSO‐d6).

### Synthesis of SATA‐PEG_2000_‐Arg

The synthesis was initiated by dissolving NH_2_‐PEG_2000_‐NH_2_ and SATA (1:1 molar ratio) in anhydrous DMF under a nitrogen atmosphere. Following 16 h magnetic stirring at ambient temperature, the crude product underwent aqueous dialysis (48 h, MWCO 3.5 kDa) prior to lyophilization, yielding the intermediate SATA‐PEG_2000_‐NH_2_. The intermediates SATA‐PEG_2000_‐NH_2_ and L‐Arginine were dissolved in MES buffer solution according to the chemical equivalent ratio of 1:1, and the reaction was stirred overnight under the condition of avoiding light, dialyzed in pure water for 48 h to remove the unreacted L‐Arginine, and lyophilized to obtain the product, SATA‐PEG_2000_‐Arg. The structures of SATA‐PEG_2000_‐Arg were verified by ^1^H NMR results (600 MHz, DMSO‐d6).

### Synthesis of DSPE‐PEG_2000_‐RGD

The RGDFK cyclic peptide (2 mg) and DSPE‐PEG_2000_‐NHS (5 mg) were dissolved in 2 mL DMF, mixed under stirring conditions, and reacted overnight at room temperature, protected from light and oxidants. The obtained solution was purified by dialysis (MWCO = 1 kDa) and freeze‐dried to obtain DSPE‐PEG_2000_‐RGD. The structure of DSPE‐PEG_2000_‐RGD was verified by ^1^H NMR results (600 MHz, DMSO‐d6).

### Preparation and Characterization of Solid Gold Nanospheres (SGN) and Hollow Gold Nanospheres (HGN)

The SGN was prepared by a two‐necked flask. In brief, 0.5 mL of 1% chloroauric acid solution and 49.5 mL of deionized water were added and heated to boiling in an oil bath at 130 degrees Celsius. After stable reflux for 10 min, 1% trisodium citrate solution was quickly added to 2 mL. After the solution became burgundy, it was continued to be heated and kept stirring for 15 min, after which the heating was stopped and the solution was cooled down to room temperature, i.e., the SGN solution was obtained.

For the HGN, 0.1mm sodium citrate, 0.1mm cobalt chloride, and 1 µm PVP were added to 100 mL pre‐deoxygenated water. Under continuous stirring, 1m sodium borohydride was added to form homogeneous cobalt nanospheres. After no bubbles were produced, 2mL 1% chloroauric acid was added to form gold shells on the cobalt nanospheres. The reaction solution was subsequently aerated, inducing oxidation of the encapsulated cobalt nanospheres. Particle sizes and polydispersity indices (PDI) of SGN and HGN were determined using a Zeta Sizer Nano‐ZS instrument (Malvern). Morphological characteristics of the SGN and HGN were analyzed by transmission electron microscopy (TEM).

### Preparation and Characterization of Solid Gold Nanomotors (SAM) and Hollow Gold Nanomotors (HAM)

The functional materials, SATA‐PEG2000‐Arg and SATA‐PEG_2000_‐Mal, were pre‐mixed with hydroxylamine hydrochloride solution and activated for 2 h at 37 °C at a molar ratio of 1:1 to deprotect SATA and expose sulfhydryl groups. Subsequently, gold nanoparticles (SGN and HGN) were immobilized on positively charged silanized glass surfaces via electrostatic adsorption. The SATA‐PEG_2000_‐Arg solution was first added and reacted overnight at room temperature. Then, the gold nanoparticles modified on one side were obtained via sonication, followed by the addition of the SATA‐PEG_2000_‐Mal solution and reaction overnight at room temperature. Finally, the modified SAM and HAM were washed with ddH_2_O by ultrafiltration centrifugation (MWCO = 10 000 Da). The particle sizes and PDI of the SAM and HAM were measured with a Zeta Sizer Nano‐ZS instrument (Malvern). The morphologies of the SAM and HAM were evaluated via TEM.

### In Vitro and In Vivo Photothermal Conversion Performances of SAM and HAM

The photothermal conversion capacity of SAM and HAM was compared under different concentrations and irradiation powers. First, SAM and HAM (1mL) at concentrations of 0.1, 0.2, 0.4, and 0.8 mg mL^−1^ were placed in flexible tubes. These samples were irradiated under 808 nm laser irradiation (1.2 W cm^−2^, 10 min). In the investigation of irradiation power impacts, SAM and HAM (1mL, 0.2 mg mL^−1^ for HAM, 0.4 mg mL^−1^ for SAM) were placed in flexible tubes and exposed to an 808 nm laser at 0.6, 1.2, and 1.8 W cm^−2^, respectively (10 min). The temperatures were recorded every 30 s with the Testo temperature measuring instrument, and the temperature profiles were plotted.

### Cumulative Release of NO

HAM and HAM‐RN after laser irradiation were incubated with hydrogen peroxide solution (200 µM). NO release was measured with a NO assay kit at 1, 2, 4, 6, 8, and 10 h to make the NO generation profiles.

### Movement Recording and Analysis

The Zetaview Twin (Particle Metrix) was used to record the motion behavior of HAMs for 9s. The motion trajectories of HAM in the H_2_O_2_ solution (HAM+H_2_O_2_), HM (HAM without SATA‐PEG_2000_‐Arg modification) in the H_2_O_2_ solution (HM+H_2_O_2_), and HAM in the ddH_2_O (HAM) were recorded. Subsequently, the tracking image sequences and the average MSD values were analyzed by MATLAB of different groups.

### Deep Tumor Penetration Assay

HAM‐Cy5.5, HM‐Cy5.5‐RN, and HAM‐Cy5.5‐RN (Cy5.5: 30 µg mouse^−1^) were injected into B16F10 tumor mouse model, respectively. At 24 h post‐injection, NIR laser irradiation (808 nm, 1.2 W cm^−^^2^, 5 min) was applied to the tumor region. Excised tumors were fixed in 4% paraformaldehyde, paraffin‐embedded, and sectioned for histological analysis. Fluorescence microscopy was employed to quantify Cy5.5 signal distribution, assessing penetration efficiency across different groups.

### In Vivo NIFR Imaging (Real‐Time Fluorescence Imaging)

After giving the injection of B16F10 cells (1 × 10^7^ cells mL^−1^, 0.1 mL) in the right axilla, the female C57BL/6J mice were divided into three groups randomly, and accepted tail vein injection with free IR780, IR780‐N, and IR780‐RN (30 µg mouse^−1^), respectively, once the tumors had grown to the appropriate size. Images were acquired using IVIS spectroscopy. Post‐euthanasia, tumors and major organs (heart, liver, spleen, lungs, and kidneys) were removed for imaging.

### In Vitro Cellular Uptake Analysis

The targeting ability of RGD to HUVEC cells was verified by a cell uptake assay. Cellular uptake was investigated by using coumarin 6 (C6) as the tracking agent, and three groups were set at the C6 concentration of 100 ng mL^−1^: Free C6, non‐targeted lipid nanoparticle containing C6 (C6‐N), and targeted lipid nanoparticle containing C6 (C6‐RN). HUVEC cells and different formulations were co‐instrumented. After 1 and 4 h, the cells were fixed by the addition of 4% histiocyte fixative, photographed by the Citation imaging reader (BioTek), and measured by flow cytometry (BD Cytoflexs).

### In Vitro Cytotoxicity Evaluation

B16F10 cells (5 × 10^3^ cells/well, 150 µL) were incubated with different concentrations of the unloaded nanoparticle (Blank), free HAM (HAM), and HAM‐RN for 48 h. The groups with photothermal therapy were exposed to 808 nm laser irradiation (1.2 W cm^−2^, 5 min) after 4 h. Subsequently, MTT solution (20 µL, 5 mg mL^−1^) and DMSO (200 µL) were added sequentially. The 96‐well plates were shaken at 37 °C for 15 min, then measured by the Citation imaging reader (BioTek) at 570 nm.

### Antigen Capture Ability of HAM‐RN

B16F10 cell lysate was mixed with PBS, HAM‐RN, and HA‐RN (HAM‐RN without Mal modification) and then incubated at 37 °C for 8h. Then, the unabsorbed proteins were removed by using dialysis bags (MWCO = 100kDa). The samples were mixed with Trypsin buffer for enzymolysis. Subsequently, the enzymatic products were examined by mass spectrometry on the Q Exactive HF‐X mass spectrometer after separated by high‐performance liquid chromatography (HPLC) and analyzed by MaxQuant 1.5.5.1.

### In Vitro Assessment of ICD Induction and DC Maturation

For ROS evaluation, B16F10s were treated with PBS, HAM, and HM (HAM without Arg modification) for 4 h. Then, cellular ROS levels were detected using DCF fluorescent probe incubation for 30 min. The HAM+L, HM‐RN+L, and HAM‐RN+L groups underwent 808 nm laser irradiation (1.2 W cm^−2^, 5 min). Fluorescence imaging was performed using an inverted microscope (Olympus), and quantitative analysis via flow cytometry. For CRT assessment, B16F10 cells were incubated with PBS, HAM, and HM, respectively. The photothermal therapy groups received identical laser parameters, with the ROS evaluation followed by 4 h incubation to enhance CRT expression. After PBS washing and 4% paraformaldehyde (4 °C, 10 min), cells were incubated with antibodies for 30 min each. The expression of CRT was determined by images captured with an inverted fluorescence microscope (Olympus) and flow cytometry. Intracellular HMGB1 analysis followed similar laser treatment steps to CRT assessment. After laser irradiation, the cells were fixed with 4% paraformaldehyde for structural preservation, followed by membrane permeabilization using 0.1% Triton X‐100 (10 min). The immunostaining protocol of intracellular HMGB1 matched CRT analysis. In addition, supernatant HMGB1 and ATP levels were quantified using ELISA and ATP assay kits, respectively.

For DC maturation, BMDCs from female C57BL/6J mice (6–8 weeks) were cultured in GM‐CSF/IL‐4‐supplemented RPMI 1640 medium (20 µg mL^−1^ each). Immature DCs in 12‐well plates were co‐cultured with treated B16F10 cells by a transwell system. Maturation markers of DCs (CD11c‐FITC, CD80‐PE, and CD86‐APC) were analyzed by flow cytometry.

### Tube Formation Assay

Mouse lymphatic endothelial cells SVEC4‐10 were placed in DMEM/F12 medium containing 0.2% FBS overnight. Subsequently, the cells were co‐incubated with Free VEGFC, VEGFC‐RN, BSA‐RN, and VEGFC‐RN+Axitinib, respectively, and added to a 96‐well plate that had been lined with 50 µL of matrix gel (2 × 10^4^ cells/well, VEGFC concentration of 100 ng mL^−1^, Axitinib concentration of 10 µg mL^−1^). Photographs were taken under the transmitted light of the microscope at 1h and 4h to evaluate the VEGFC‐induced tube formation, and processed semi‐quantitatively with Image J.

### In Vivo Lymphatic Vessel Reconstructing at the Tumor Site

Free VEGFC, VEGFC‐RN, BSA‐RN, and VEGFC‐RN+Axitinib were injected into the B16F10‐bearing mice via the tail vein, respectively (VEGFC: 3 µg mouse^−1^; HAM: 1 mg/kg). After 2 days, the mice were injected with HAM‐RN with Mal replaced by Cy5.5 and received 808nm laser irradiation (1.2W cm^−2^, 5min) after 12 h. The mice were euthanized a day later, the tumors and LNs were obtained, and analyzed by NIFR imaging.

### Antitumor Activity in B16F10 and 4T1 Tumor Mouse Models

The antitumor effects of V+HAM‐RN+L were evaluated on the primary B16F10 melanoma mouse model and the primary 4T1 triple‐negative breast tumor mouse model. 6–8 weeks’ female C57BL/6J mice bearing 100mm^3^ B16F10 tumors and 6–8 weeks’ female BALB/c mice bearing 100mm^3^ 4T1 tumors were assigned into 9 groups randomly (n = 5), which were injected with the following formulations: (1)Normal saline (NS), (2) HAM‐RN, (3) HM‐RN+Laser (HM‐RN+L), (4) HAM‐N+Laser (HAM‐N+L), (5) HAM‐RN+Laser (HAM‐RN+L), (6) HAM+Laser (HAM+L), (7) VEGFC‐RN, (8) HM‐RN+VEGFC‐RN+Laser (HM‐RN+VEGFC‐RN+L), (9) HAM‐RN+VEGFC+Laser (HAM‐RN+VEGFC+L). The dose of HAM was 1 mg kg^−1^ and the dose of VEGFC was 2ug mouse^−1^. On the day after receiving the injection, the photothermal therapy groups received laser irradiation (808 nm, 1.2 W cm^−2^, 5 min). Every three days was a treatment cycle. The tumor volumes of the mice were measured by a vernier caliper, and the body weights were recorded every two days. For animal ethics, mice were euthanized when the tumor volume reached 2000 mm^3^. On the 16th day of treatment, mice were euthanized, tumors were obtained, and tumors were weighed and imaged.

### In Vivo Anti‐Metastasis Assessment

The same grouping strategy was adopted for the metastasis‐resistant mouse model as for the primary mouse model. When the volumes of B16F10 axillary load tumor reached 100 mm^3^, B16F10 cells (2 × 10^6^ cells/mouse) were administered with tail vein injection. The treatment protocol was the same as the In vivo antitumor therapy. After 16‐day treatment, the mice were euthanized, and the lungs were isolated, photographed, and the number of lung nodules was assessed.

### In Vivo Immunization Study

Following in vivo evaluation of antitumor effects, the excised tumors, spleens, and TDLNs of mice were obtained, sheared with surgical scissors, and milled through 200 mesh copper networks to generate single‐cell suspensions. Then, lymphocytes were separated by a 40% Percoll solution. T cells, CTLs, and Tregs in tumor tissues, DCs in TDLN of the primary model, and memory T cells in spleens of the metastasis‐resistant model were detected by flow cytometry (BD Cytoflexs) and analyzed by FlowJo software. In addition, cytokine levels of TNF‐α, IFN‐γ, IL‐10, and TGF‐β were determined by an Elisa kit collection of supernatants from fragmented tumor tissue.

### Immunohistochemical Analysis

Murine major organs underwent 4% paraformaldehyde fixation followed by paraffin embedding, sectioning, and histopathological assessment via hematoxylin‐eosin (H&E) staining. Tumor specimens were subjected to proliferation/apoptosis analysis using Ki67 immunostaining and TUNEL assays. Reprogramming of tumor‐associated blood and lymphatic vessels was observed by LYVE‐1, CD31, NG2, HIF‐α, ZBTB46, CD4, CD8, and DAPI staining of tumor sections.

### Statistical Analysis

GraphPad Prism 8.0.1 and Microsoft Excel 2021 were used in this work for statistical analysis. All data were expressed as the mean±SD. For multiple groups, one‐way ANOVA analysis of variance with Tukey's post hoc test was used for analysis of statistical significance. ^*^ *p* < 0.05, ^**^ *p* < 0.01, ^***^ *p* < 0.001 were considered statistically significant, and ns (*p*>0.05) was considered no significant difference.

## Conflict of Interest

The authors declare no conflict of interest.

## Supporting information



Supporting Information

Supplemental Movie 1

Supplemental Movie 2

Supplemental Movie 3

## Data Availability

The data that support the findings of this study are available in the supplementary material of this article.
