# Supplementary material for: NO‐Driven Janus Nanomotor Enhances T‐Cell Infiltration by Reconstructing Tumor‐Associated Blood and Lymphatic Vessels
Source: Adv Sci (Weinh). 2025 Sep 8;12(44):e12090. doi: 10.1002/advs.202512090 (PMC12667450; doi:10.1002/advs.202512090)
Supplement: Supplementary file 1 — Supporting Information [file ADVS-12-e12090-s003.docx]

Supporting Information

**NO-Driven Janus Nanomotor Enhances T-cell Infiltration by Reconstructing Tumor-associated Blood and Lymphatic Vessels**

Qingping Ma^#^, Shunli Fu^#^, Yiming Xia, Shuying Gao, Zhenxing Xia, Panpan Gu, Shijun Yuan, Jinhu Liu, Shuang Liang, Nan Li, Qinglin Yang, Weiwei Mu, Jie Liu, Xinrui Liu, Yongjun Liu* and Na Zhang*

^#^The authors contributed equally to this work.

*Corresponding author.

E-mail: liuyongjun@sdu.edu.cn (Yongjun Liu); zhangnancy9@sdu.edu.cn (Na Zhang).

Department of Pharmaceutics, Shandong Key Laboratory of Targeted Drug Delivery and Advanced Pharmaceutics, NMPA Key Laboratory for Technology Research and Evaluation of Drug Products, Key Laboratory of Chemical Biology (Ministry of Education), State Key Laboratory of Discovery and Utilization of Functional Components in Traditional Chinese, School of Pharmaceutical Sciences, Cheeloo College of Medicine, Shandong University, 44 Wenhuaxi Road, Jinan, Shandong Province 250012, China

**
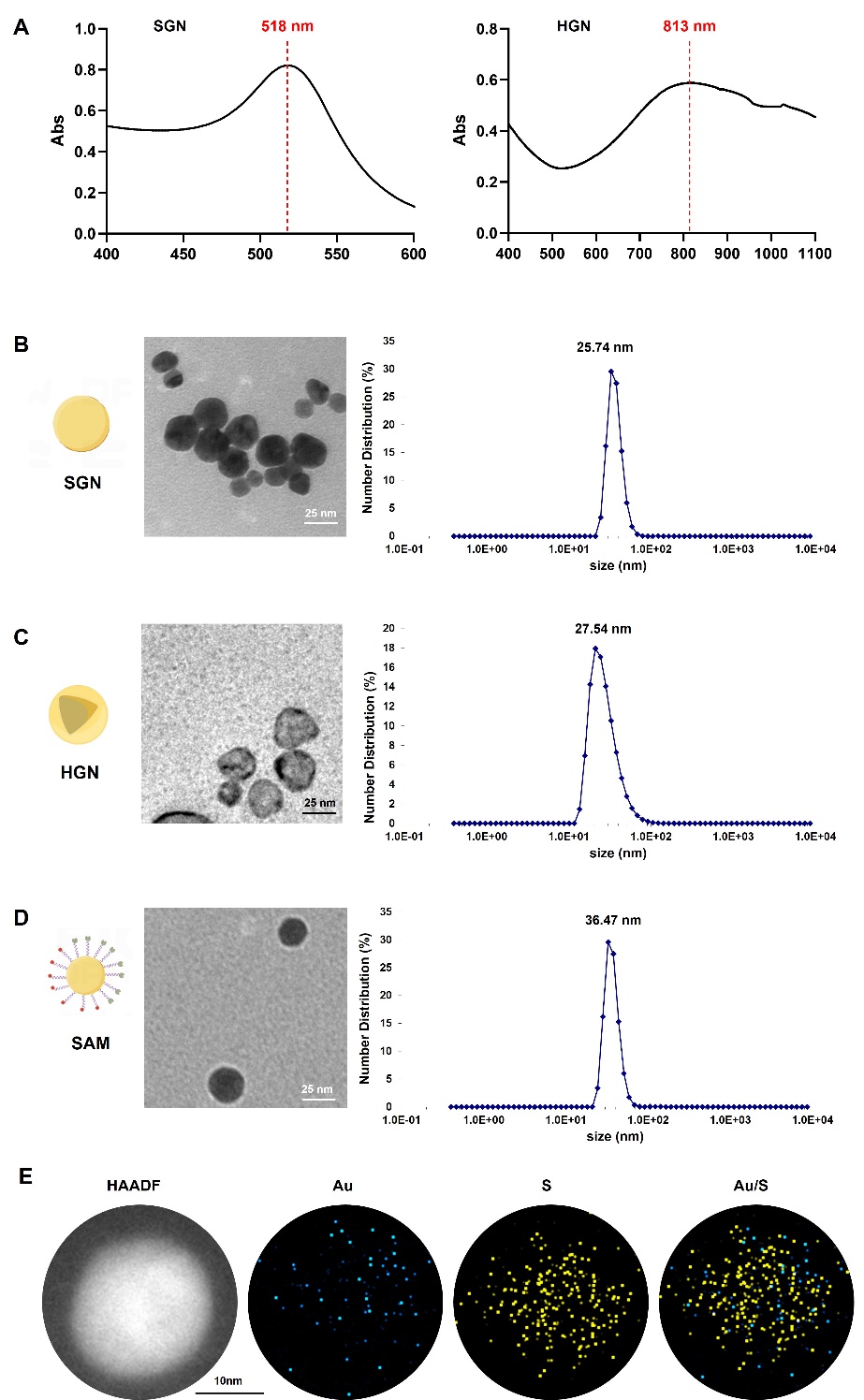
**

**Figure S1.** Characterization of SGN, HGN and SAM. **A,** UV-VIS absorption spectra results of the SGN and HGN. **B-D,** The transmission electron microscope image and particle size of SGN **(B)**, HGN **(C)** and SAM **(D)**. **E,** STEM-EDS elemental mapping analysis of SAM.


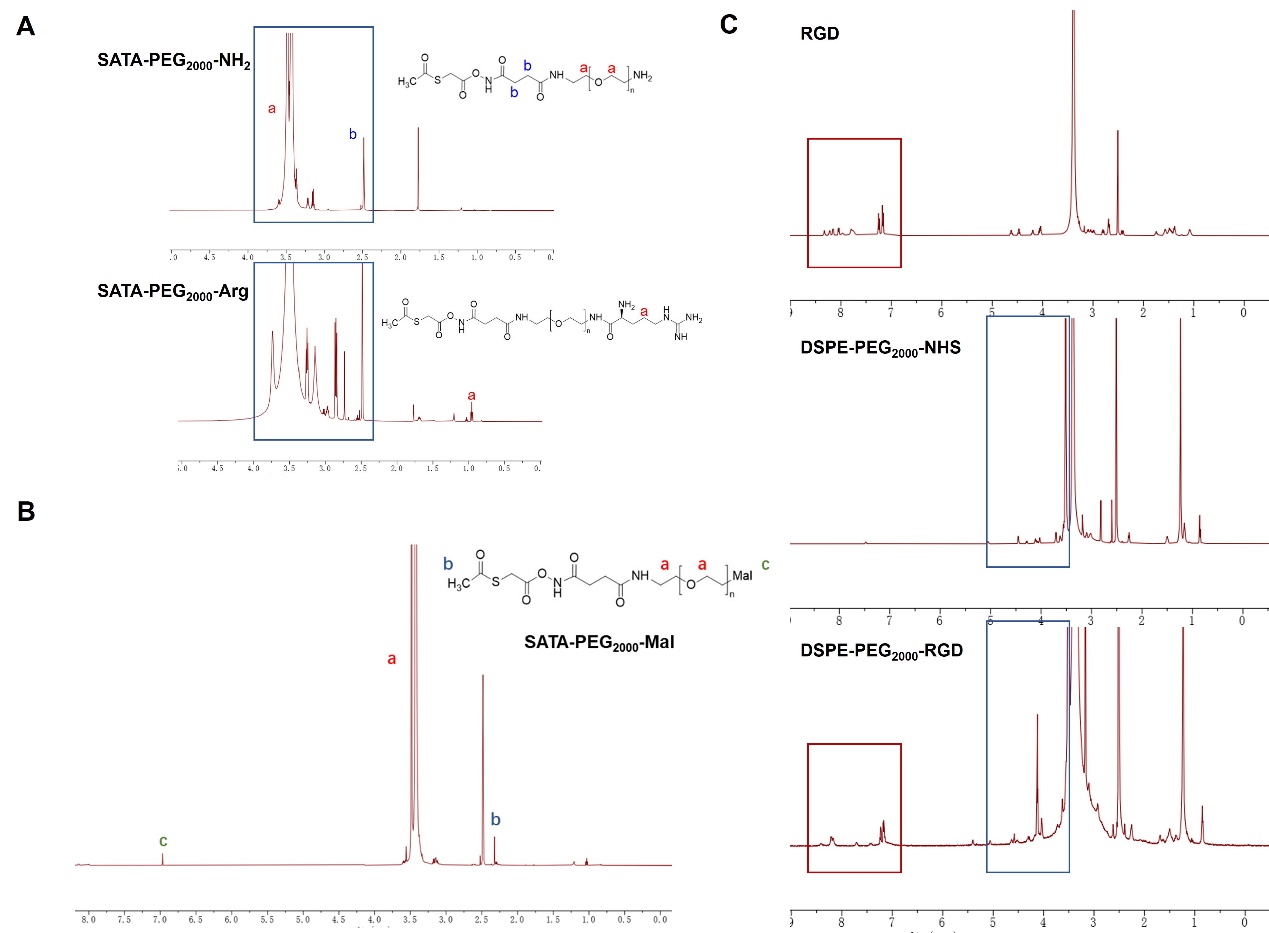


**Figure S2.** Characterization of **A,** SATA-PEG_2000_-NH_2_, SATA-PEG_2000_-Arginine (SATA-PEG_2000_-Arg), **B,** SATA-PEG_2000_-Maleimide (SATA-PEG_2000_-Mal) and **C,** Cyclo-RGDfK (RGD), DSPE-PEG_2000_-NHS and DSPE-PEG_2000_-RGD.


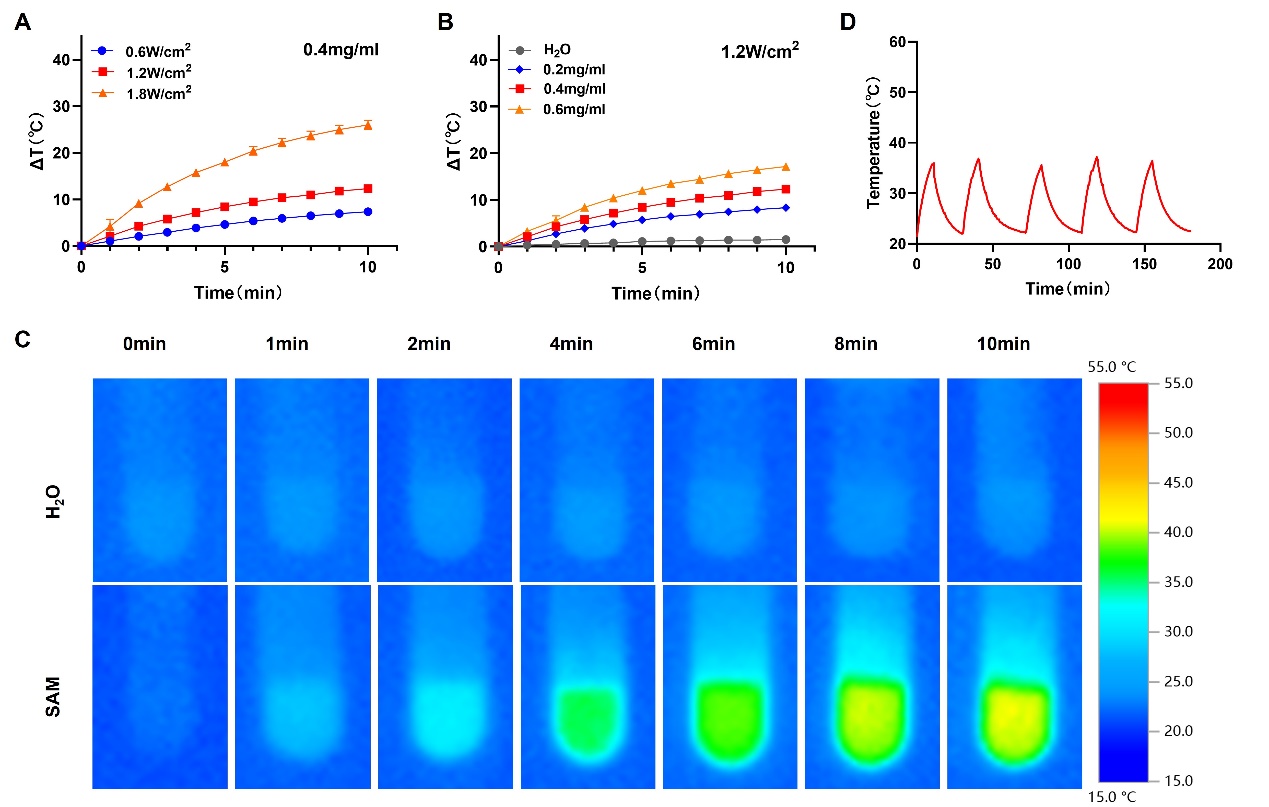


**Figure S3.** The evaluation of the photothermal conversion capability of SAM. **A,** Temperature profiles of SAM (0.4 mg/mL) with 808 nm laser irradiation at 0.6 W/cm^2^, 1.2 W/cm^2^ and 1.8 W/cm^2^. (n = 3 biologically independent experiments). **B,** Temperature profiles of SAM of 0, 0.2, 0.4 and 0.6 mg/ml after irradiation with 808 nm laser irradiation at 1.2 W/cm^2^. (n = 3 biologically independent experiments). **C,** Infrared thermal imaging of water and SAM at different time points after irradiation with a laser irradiation (808 nm) at 1.2 W/cm^2^ for 10 min. **D,** Temperature profile of SAM recorded under five cycles of the laser (808 nm) on/off.


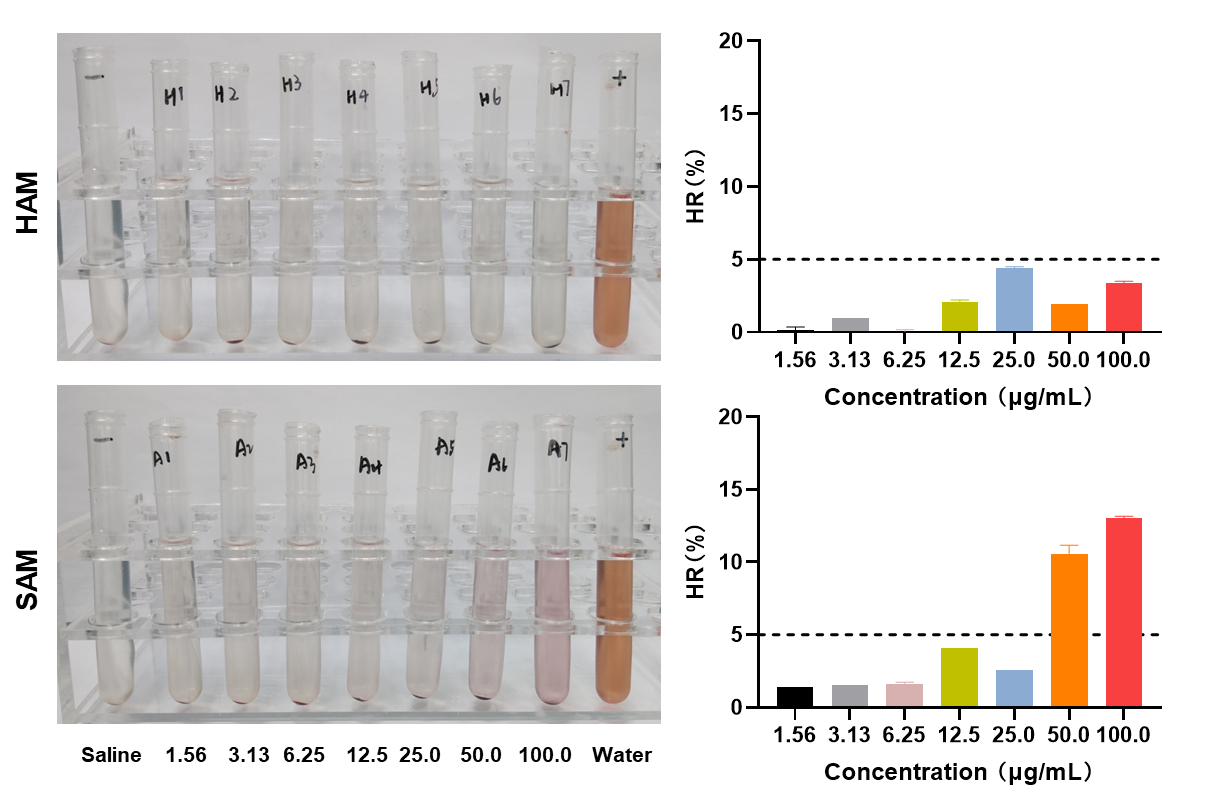


**Figure S4.** The photographs and haemolysis rate analysis of the haemolysis test for HAM and SAM (n = 3 biologically independent experiments).


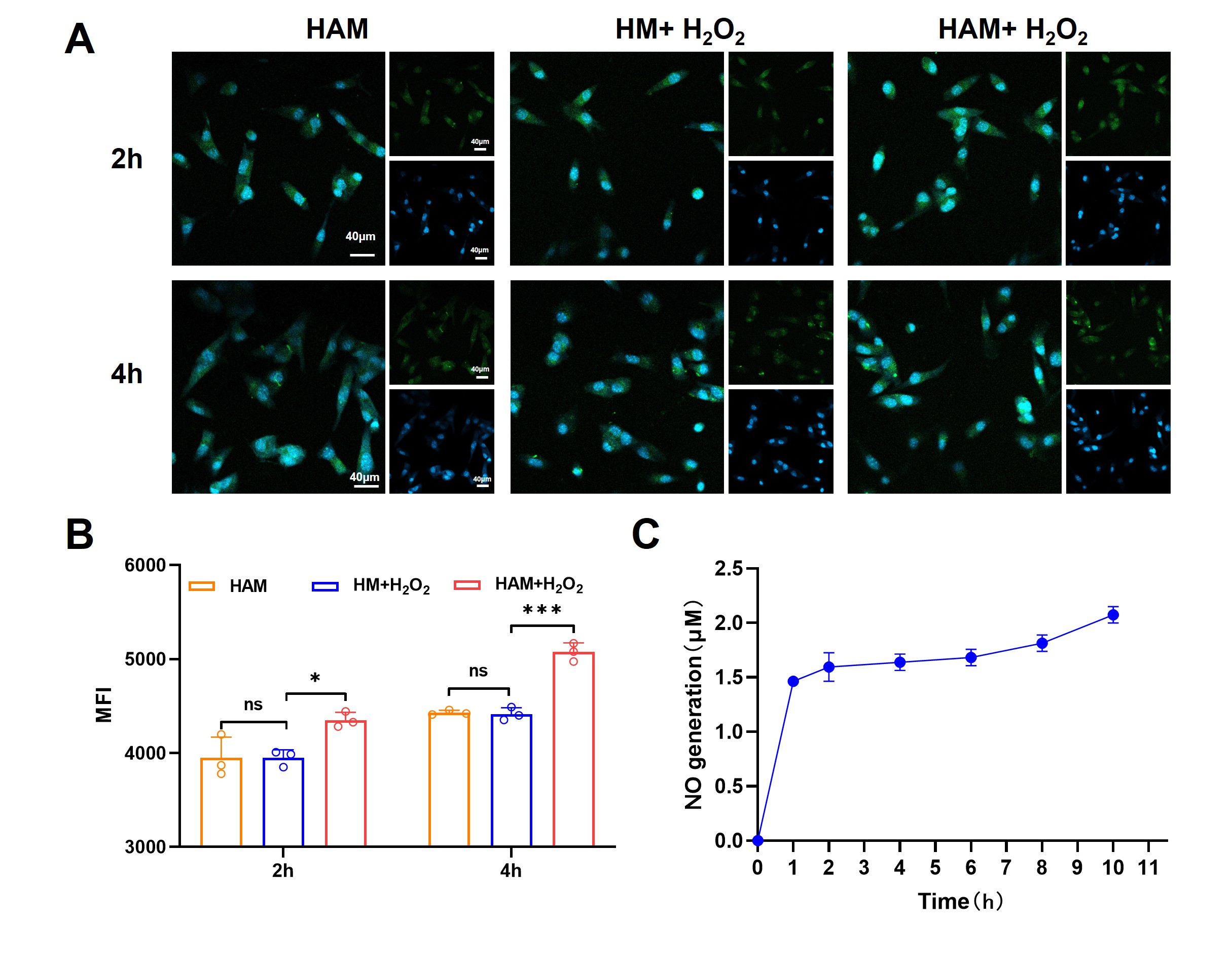


**Figure S5.** **A-B,** Confocal fluorescence images and fluorescence intensities measured via flow cytometry of B16F10 cells incubated with HAM, HM+H_2_O_2_ and HAM+ H_2_O_2_ after 2 h and 4 h. Scale bar: 40 μm. **C,** The generation profile of NO from HAM in H_2_O_2_ solution during 10 hours. (n = 3 biologically independent experiments). Statistical significance was calculated by one-way ANOVA analysis of variance with Tukey’s post hoc test. ns: no significance, *p*>0.05, **p*<0.05, ****p*<0.001.


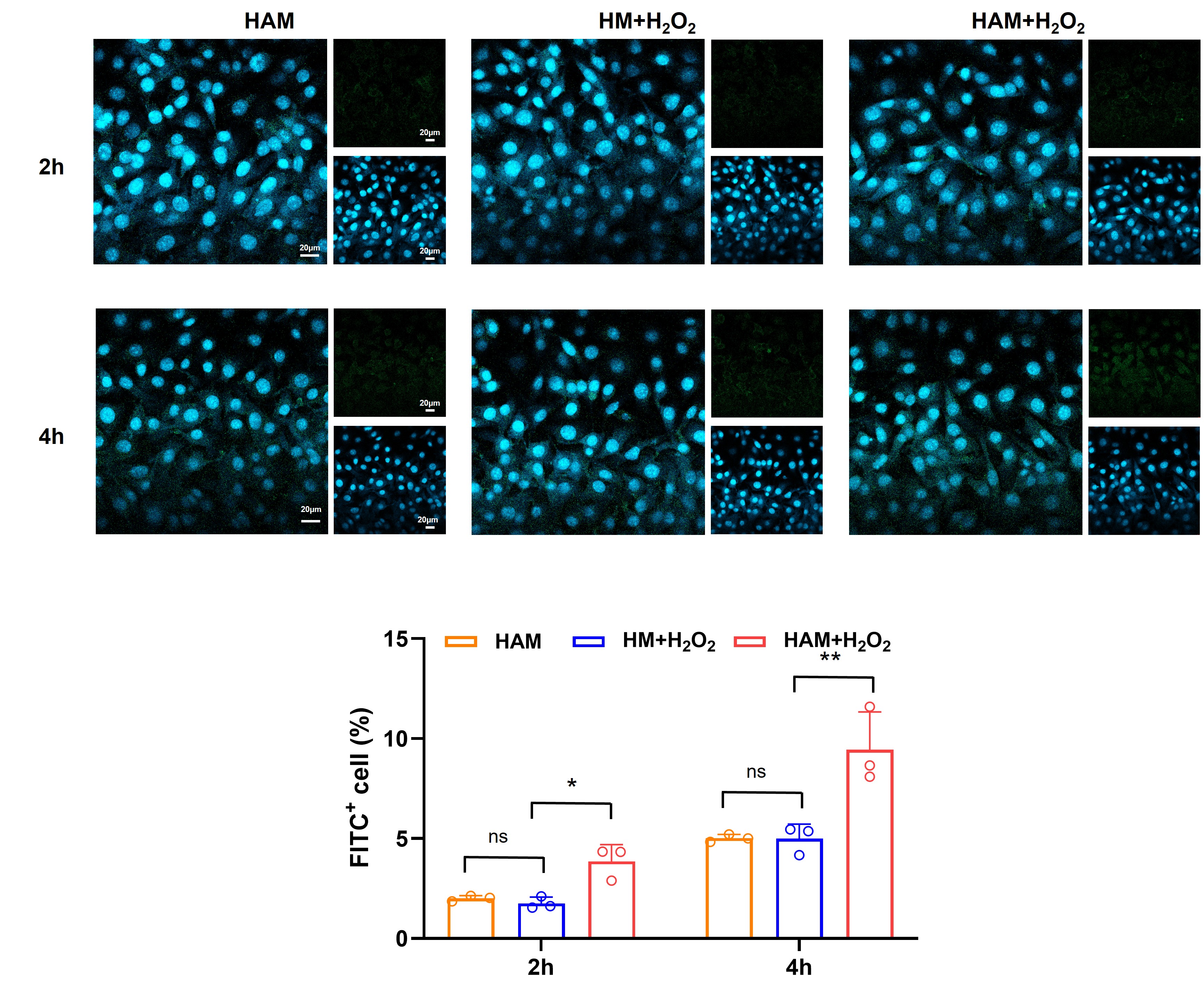


**Figure S6.** Confocal fluorescence images and fluorescence intensities measured via flow cytometry of B16F10 cells incubated with HAM, HM+H_2_O_2_ and HAM+ H_2_O_2_ in ECM-mimicking Transwell system after 2 h and 4 h. Scale bar: 20 μm. Statistical significance was calculated by one-way ANOVA analysis of variance with Tukey’s post hoc test. ns: no significance, *p*>0.05, **p*<0.05, ***p*<0.01.


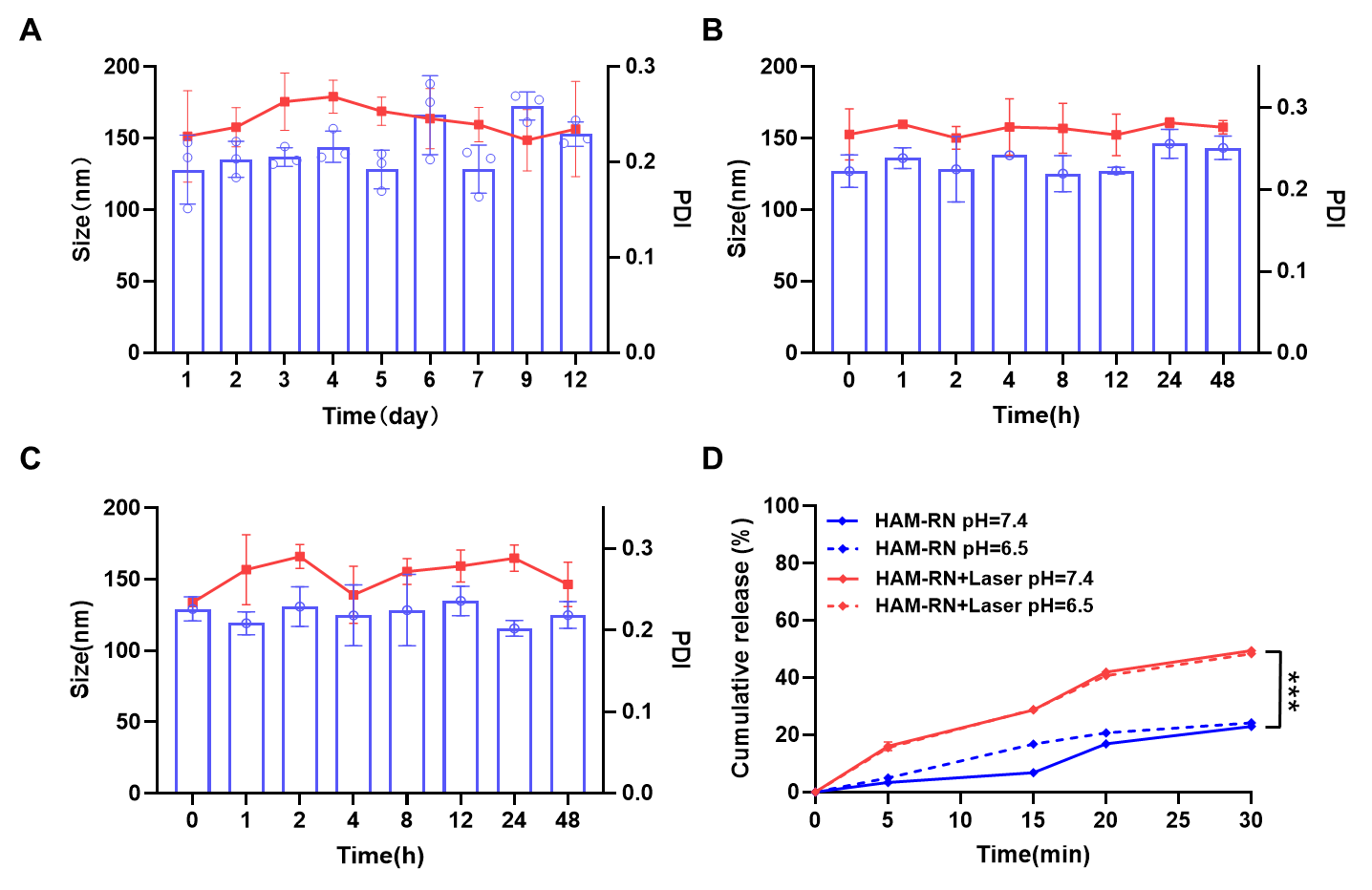


**Figure S7. A,** The storage stability of HAM-RN. **B,** The stability in cell culture medium of HAM-RN. **C,** The stability in serum of HAM-RN. **D,** The release profiles of HAM-RN. Statistical significance was calculated by one-way ANOVA analysis of variance with Tukey’s post hoc test. ns: no significance, ****p* < 0.001.


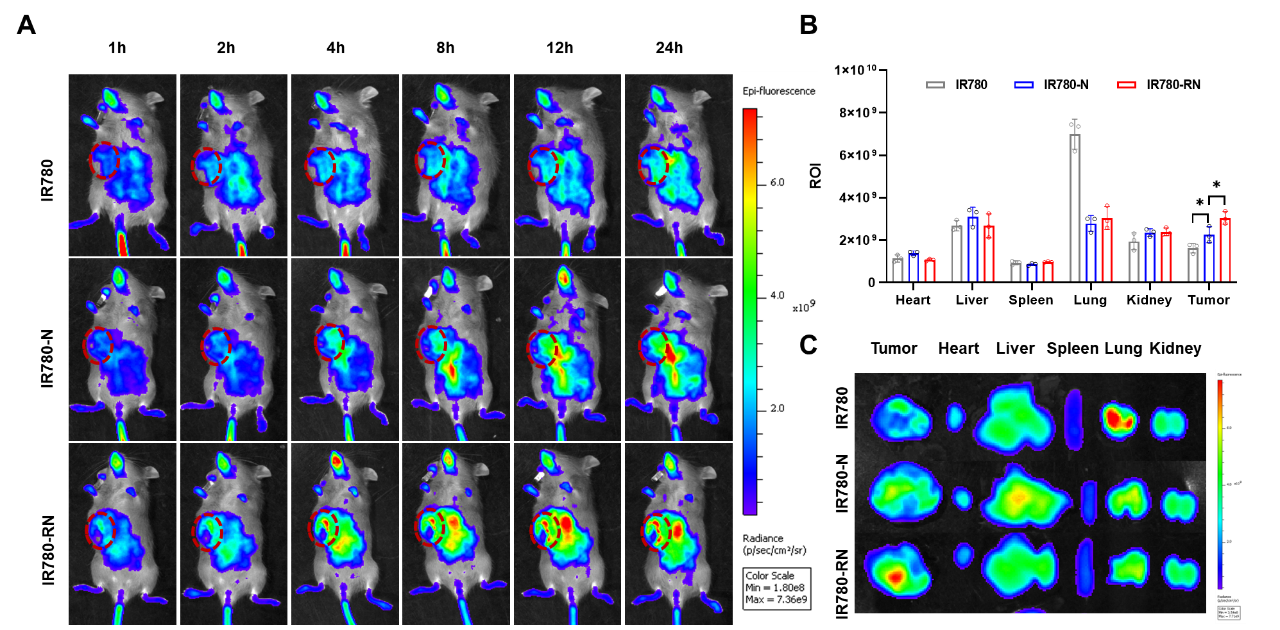


**Figure S8.** **A,** *In vivo* biodistribution imaging of the mice after injection of IR780, IR780-N and IR780-RN, respectively (i.v.). **B,** Average fluorescence intensities of the tumor and the main organs (including the heart, liver, spleen, lung and kidney) at 48 h. **C,** *Ex vivo* imaging of the tumor and the main organs (including the heart, liver, spleen, lung and kidney) at 48 h. Statistical significance was calculated by one-way ANOVA analysis of variance with Tukey’s post hoc test. **p*<0.05.


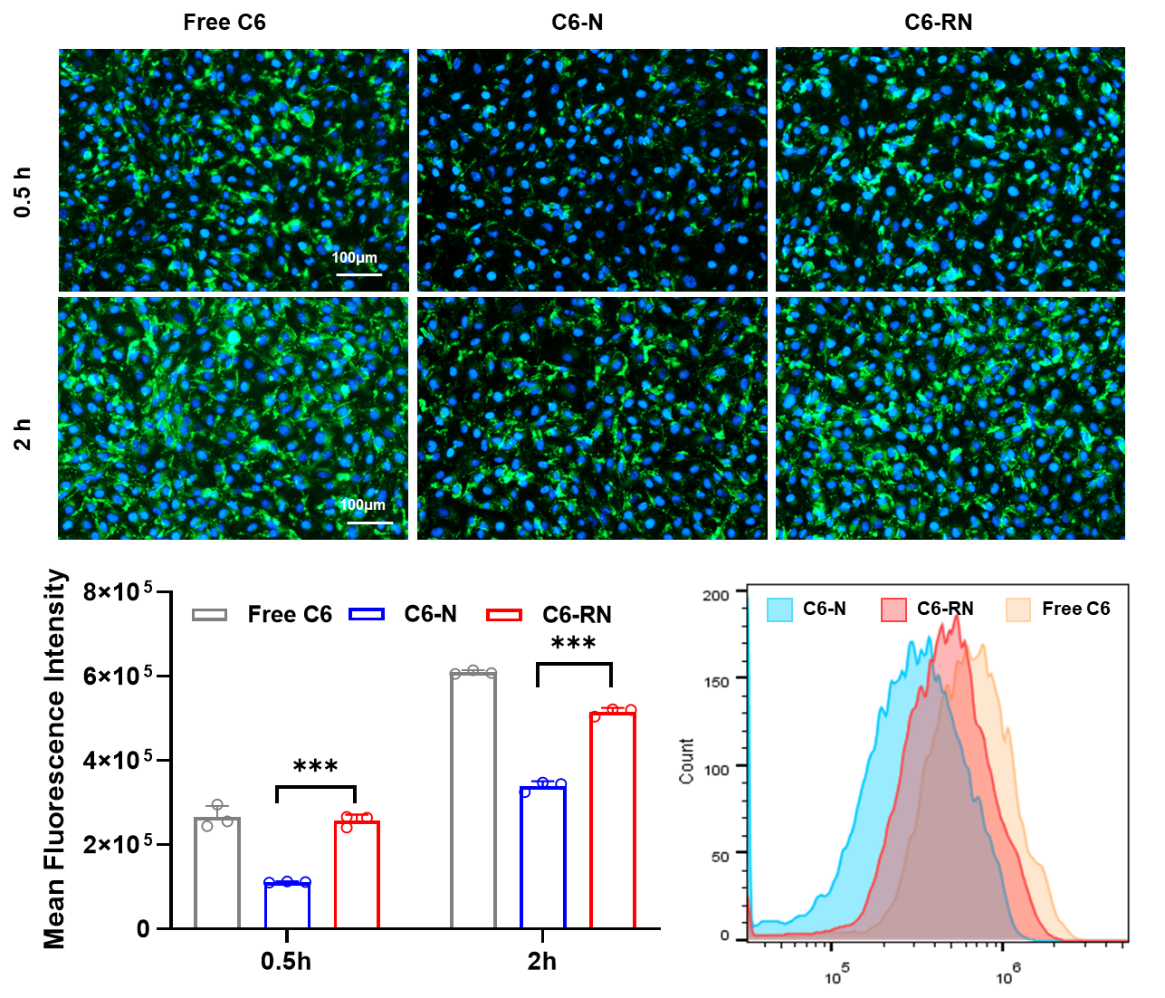


**Figure S9.** Representative images obtained with fluorescence microscopy and fluorescence intensities of HUVEC cellular uptake after treating with free C6, C6-N and C6-RN, respectively. Scale bar: 100 μm. Statistical significance was calculated by one-way ANOVA analysis of variance with Tukey’s post hoc test. ****p*<0.001.


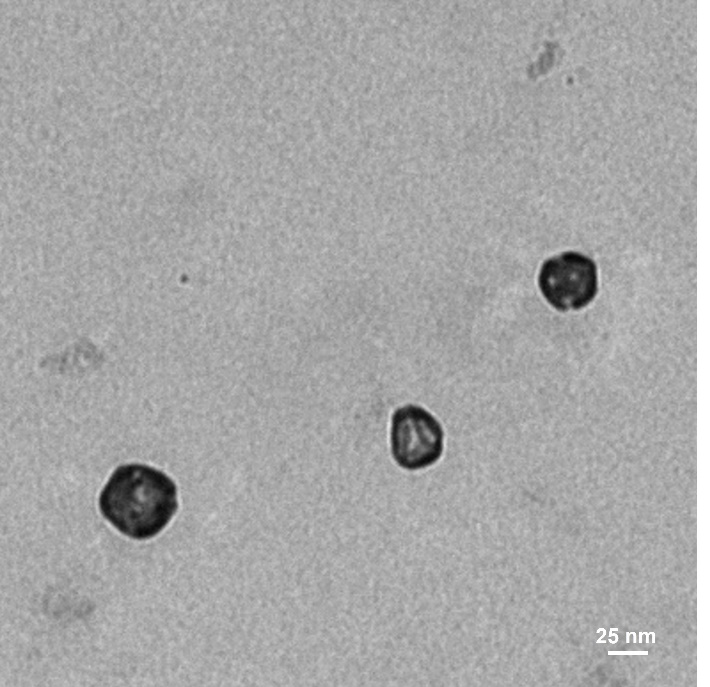


**Figure S10.** Morphology of HAM-RN after 808 nm laser irradiation.


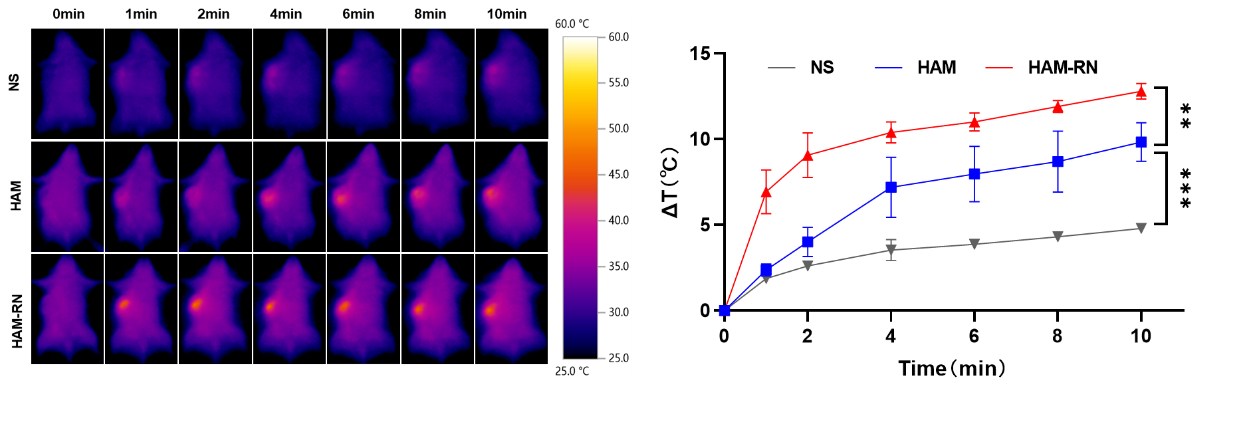


**Figure S11.** Infrared thermal images and the temperature profiles of tumor regions of B16F10 tumor-bearing mice injected with NS, HAM and HAM-RN under 808 nm laser irradiation (1.2W/cm^2^, 10 min). Statistical significance was calculated by one-way ANOVA analysis of variance with Tukey’s post hoc test. ***p*<0.01, ****p*<0.001.


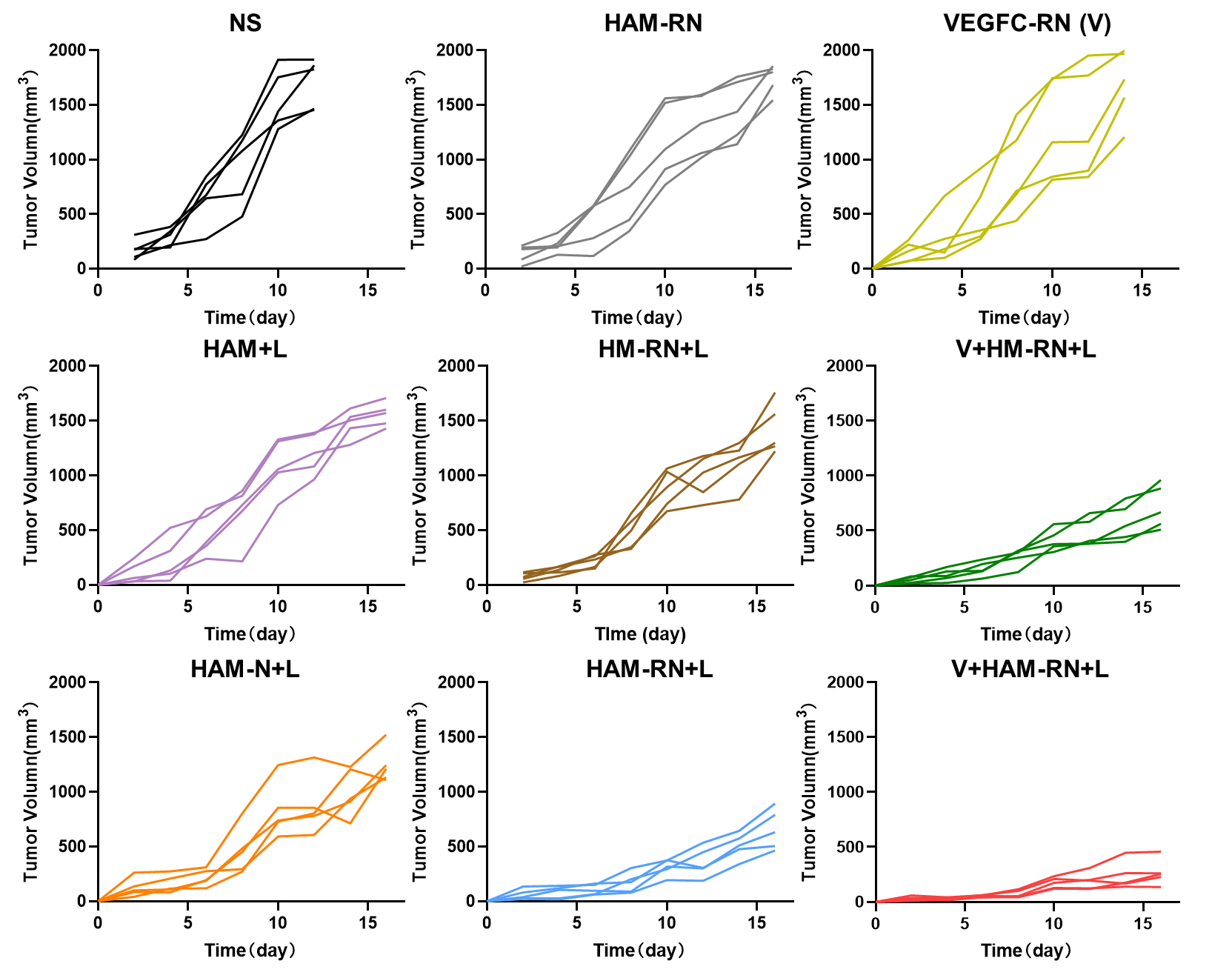


**Figure S12.** The B16F10 individual tumor volume curves in NS, HAM-RN, VEGFC-RN (V), HAM+L, HM-RN+L, V+HM-RN+L, HAM-N+L, HAM-RN+L and V+HAM-RN+L (n=5 biologically independent mice each group).


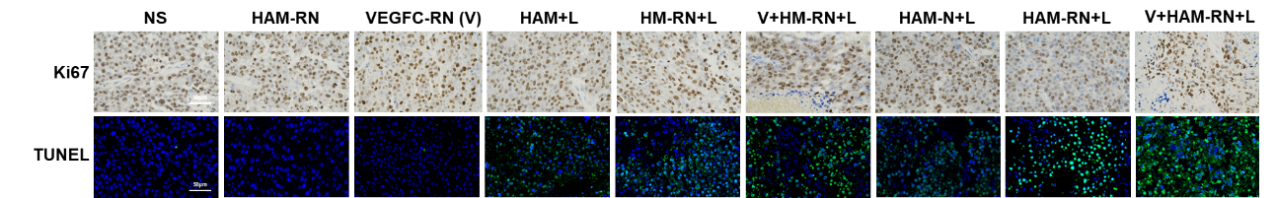


**Figure S13.** The Ki67 and TUNEL staining of tumor tissues in the B16F10-bearing mouse model. Scale bar: 50 μm.


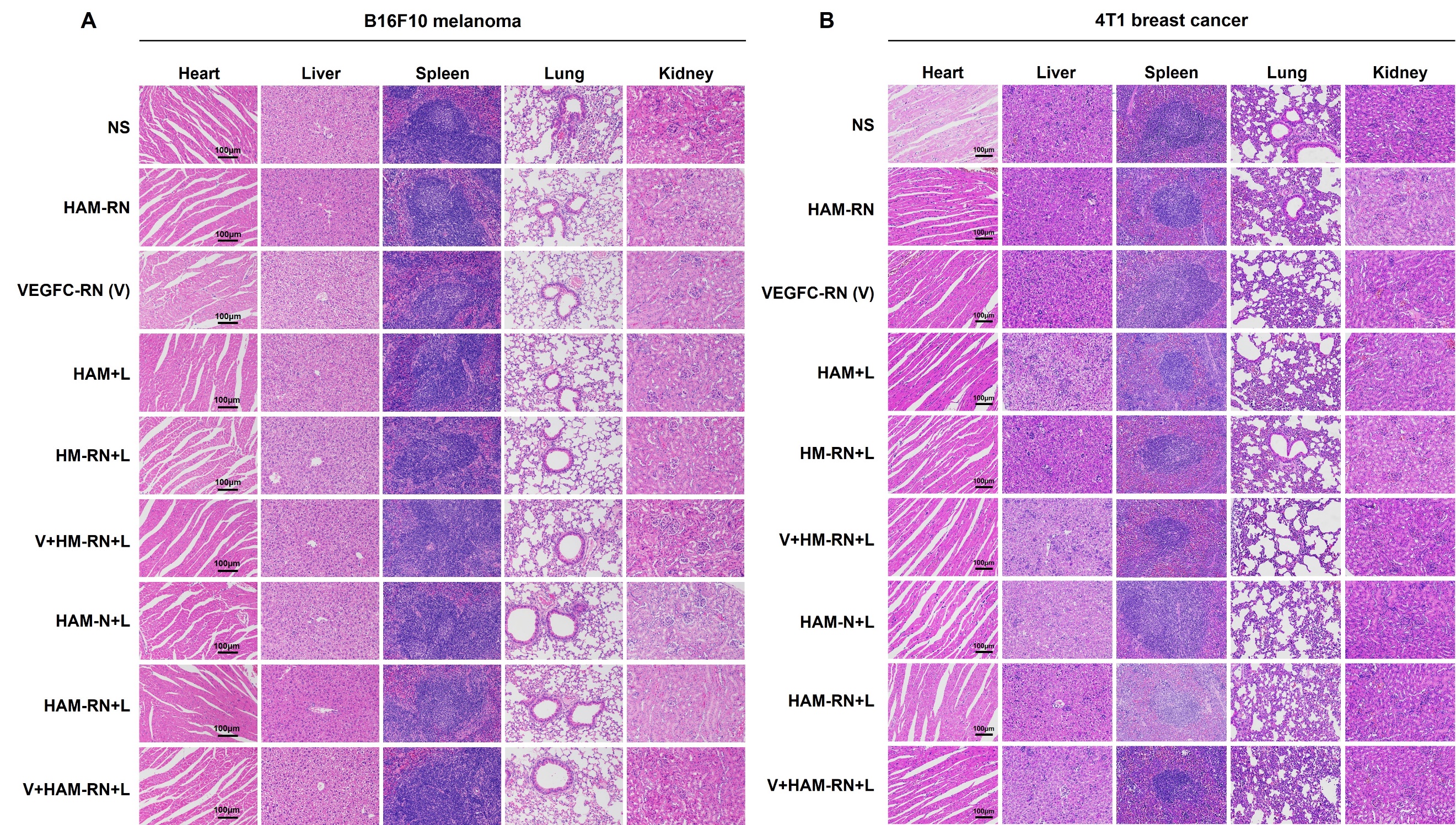


**Figure S14.** The H&E staining of heart, liver, spleen, lung and kidneys from **A,** B16F10-bearing mice and **B,** 4T1-bearing mice with different formulations treatment. Scale bar: 100 μm.


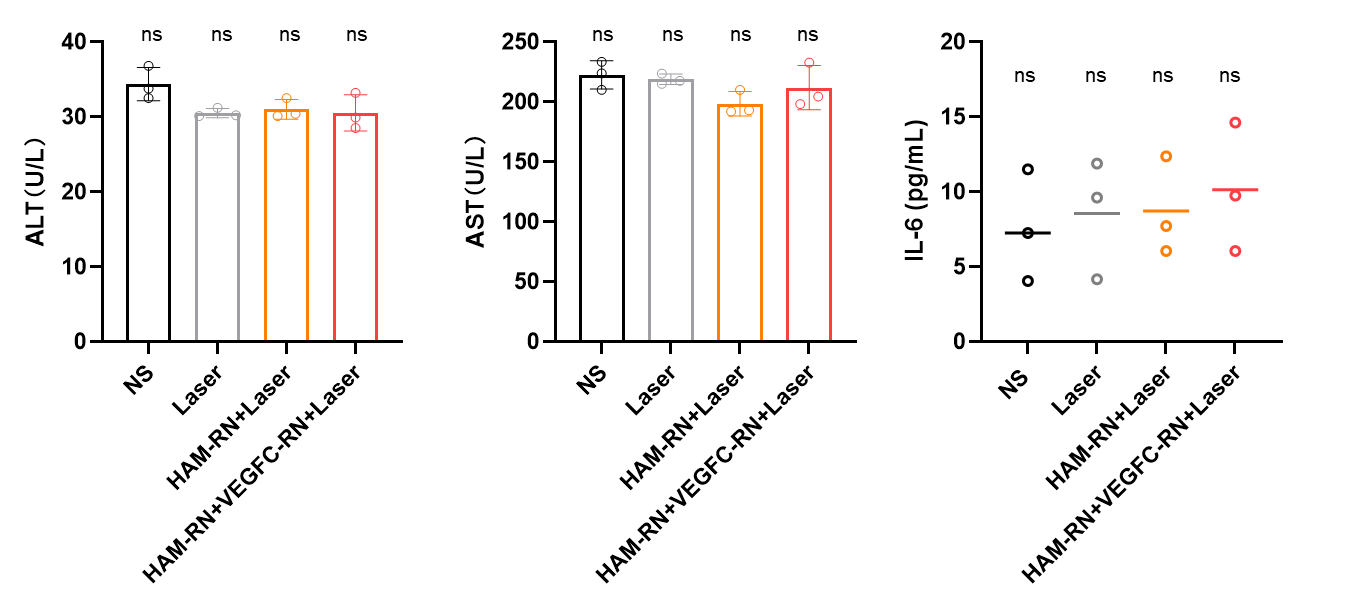


**Figure S15.** The AST, ALT and IL-6 levels in the serum of mice treated with different formulations. All experiments were conducted with three independent replicates. Results are expressed as the mean ± SD. One-way analysis of variance (ANOVA) with Tukey’s post hoc test was used to assess significance. ns: no significance.


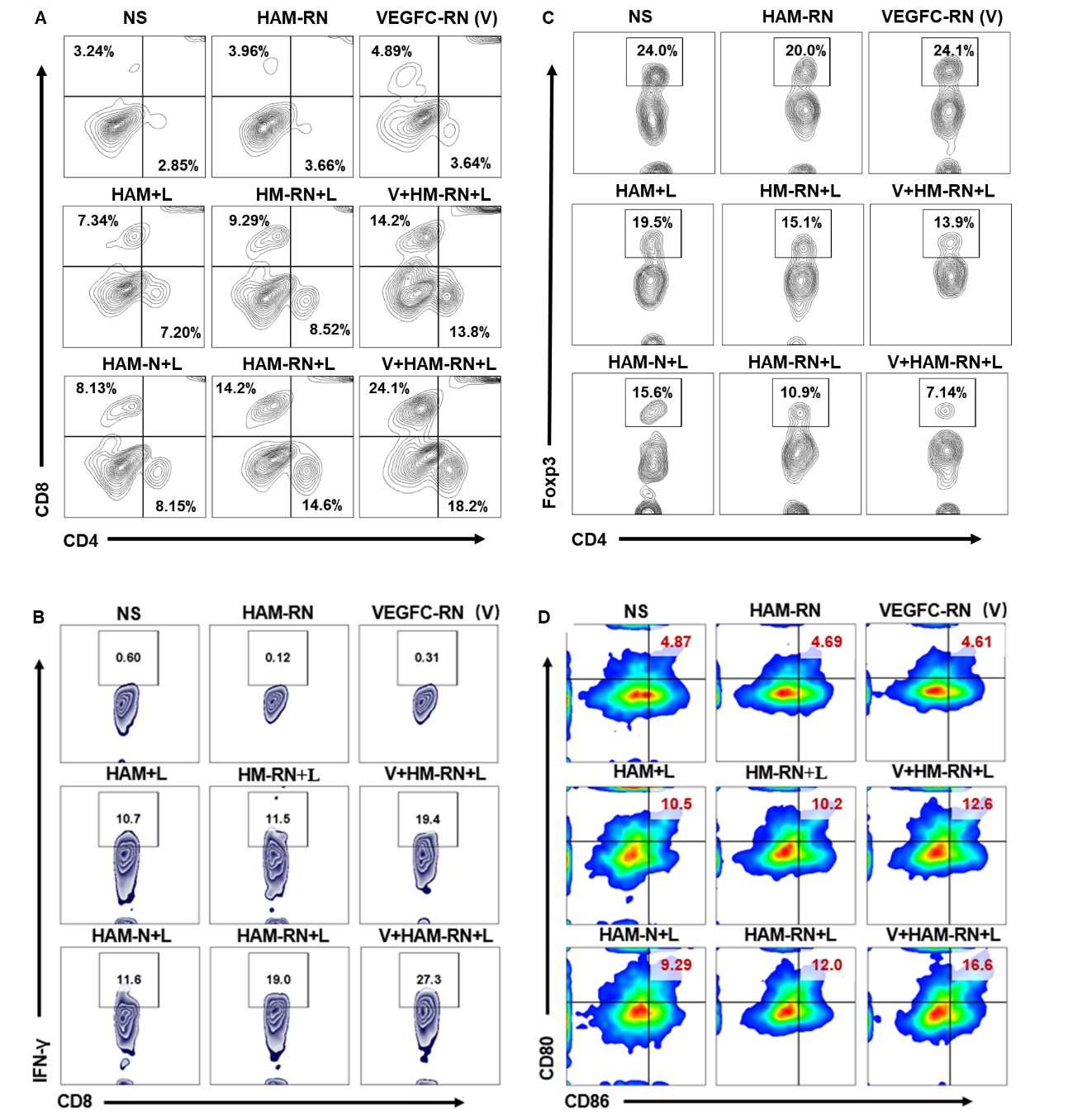


**Figure S16.** The representative flow cytometry image of **A,** CD4^+^ T and CD8^+^ T cells, **B,** CTLs (CD8^+^IFN-γ^+^T cells), **C,** Tregs (CD4^+^Foxp3^+^T cells) and **D,** mature DCs (CD11c^+^CD80^+^CD86^+^ cells) in the B16F10 tumor-bearing mouse model.


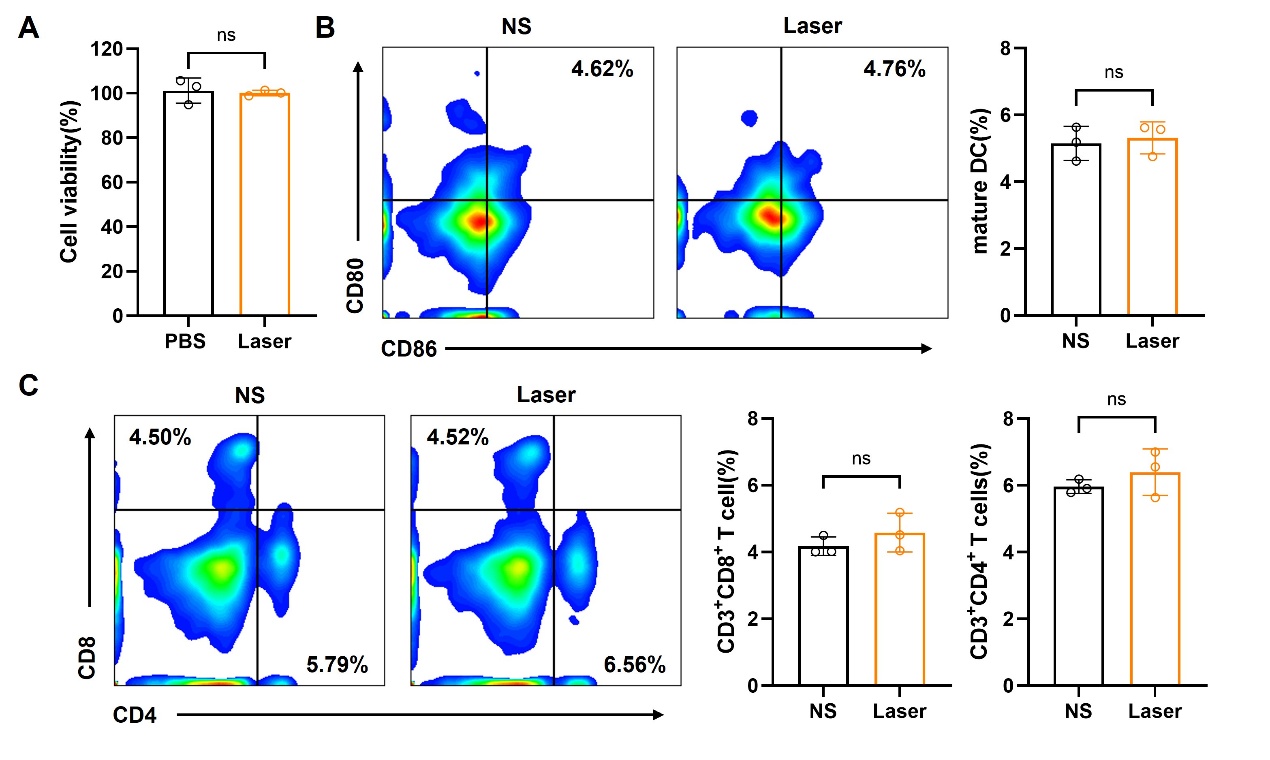


**Figure S17.** The independent effects of laser-induced local heating to immune and cellular responses. **A,** B16F10 cell viabilities in the PBS and Laser groups. **B,** Flow cytometric quantification of mature DCs in TDLNs. **C,** Flow cytometric quantification of intratumoral CD4^+^ T and CD8^+^ T cells. All experiments were conducted with three independent replicates. Results are expressed as the mean ± SD. One-way analysis of variance (ANOVA) with Tukey’s post hoc test was used to assess significance. ns: no significance.


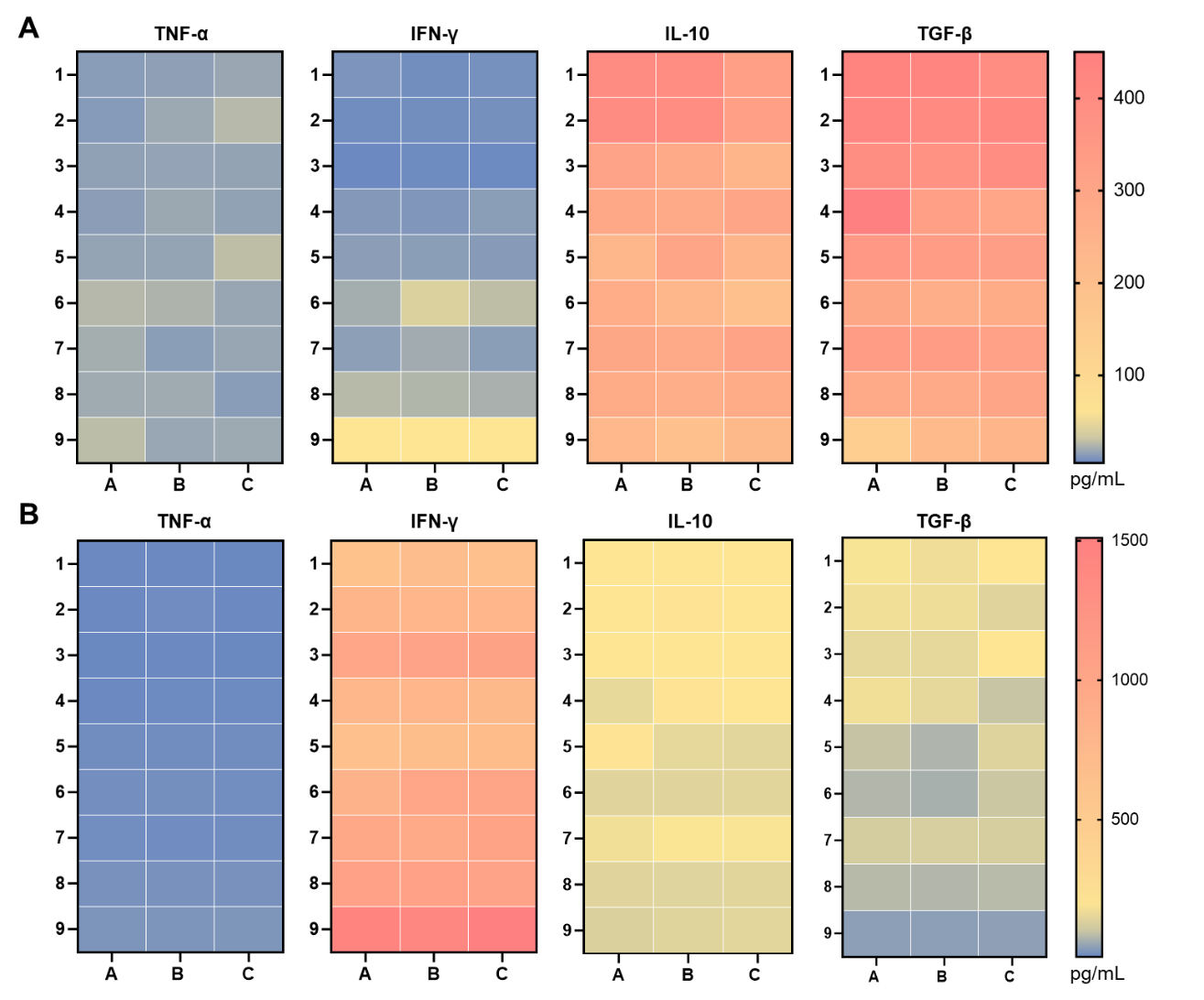


**Figure S18.** The heat map of intratumoral levels of TNF-α, IFN-γ, IL-10, TGF-β after treatment with different formulations in A, the B16F10-bearing mouse model and B, the 4T1-bearing mouse model.


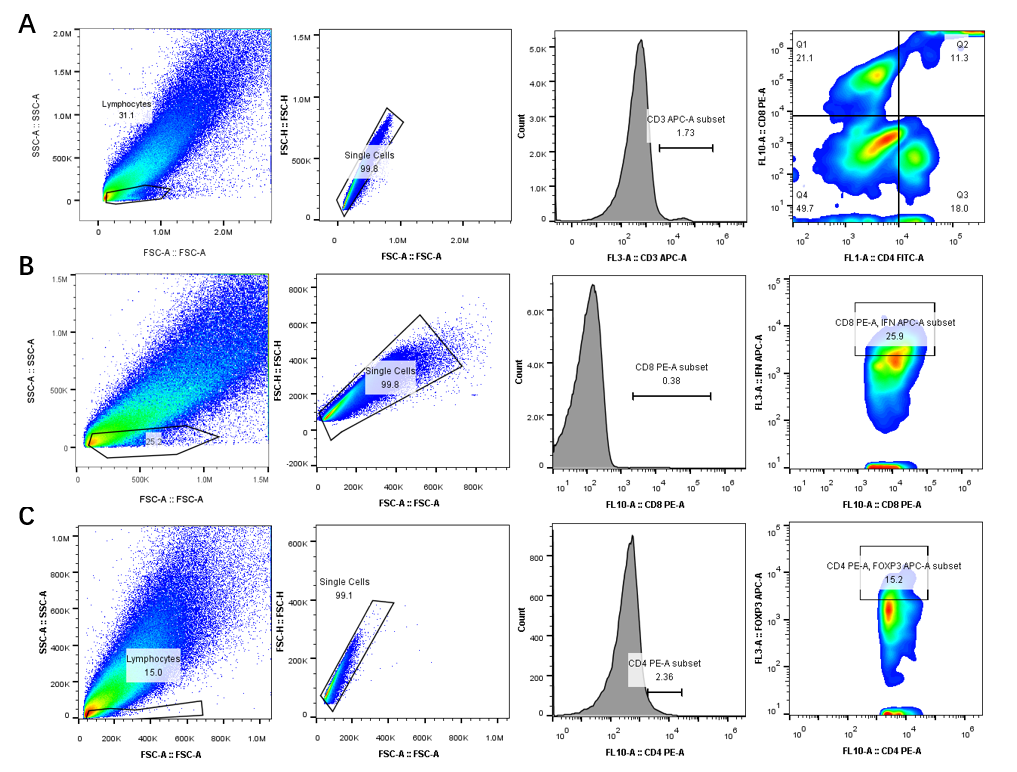


**Figure S19.** Gating strategies for different types of T cells in tumor. **A,** Gating strategy for CD4^+^T (CD3^+^CD4^+^) cells and CD8^+^T (CD3^+^CD8^+^) cells. **B,** Gating strategy for CTLs (CD8^+^IFN-γ^+^). **C,** Gating strategy for Treg cells (CD4^+^Foxp3^+^).


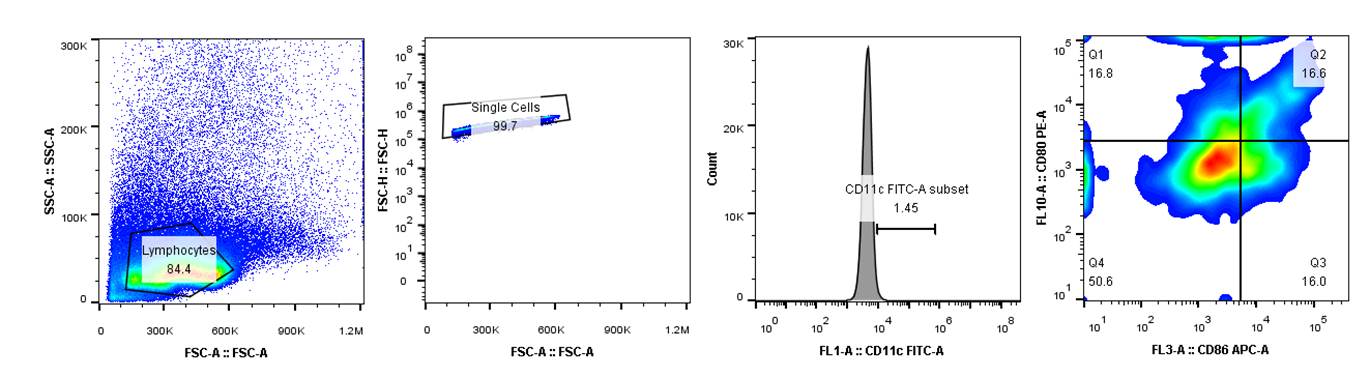


**Figure S20.** Gating strategies for mature DCs (CD11c^+^CD80^+^CD86^+^) in tumor draining lymph nodes.


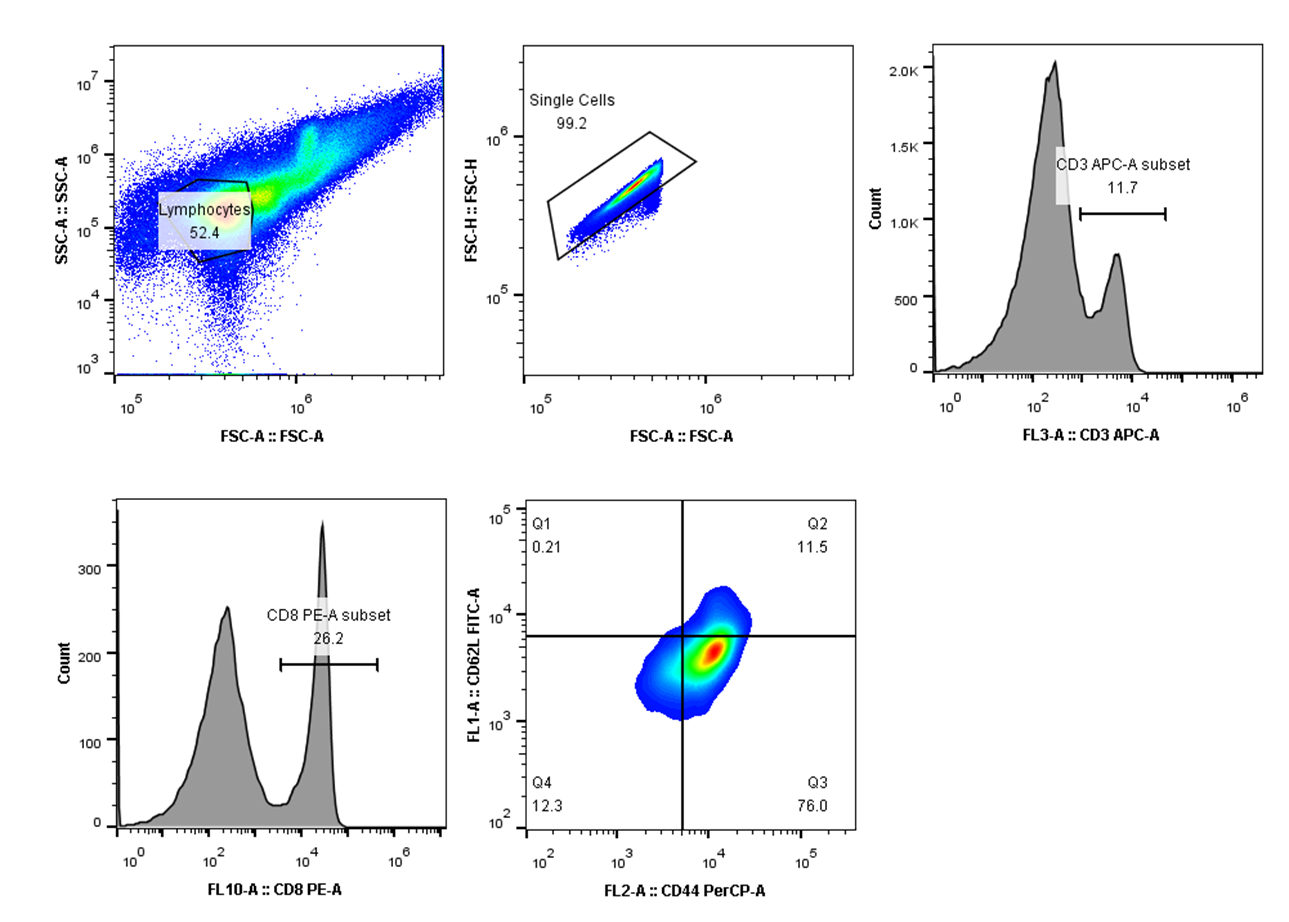


**Figure S21.** Gating strategies for central memory T cells (T_CM_, CD3^+^CD8^+^CD44^+^CD62L^+^) and effector memory T cells (T_EM_, CD3^+^CD8^+^CD44^+^CD62L^-^) in spleen.
